# Sphingolipid metabolism and hematologic disorders: current understanding and future directions

**DOI:** 10.3389/fphys.2026.1826317

**Published:** 2026-05-29

**Authors:** Zheshu Kuang, Tianjun Huang, Guanjun Chen

**Affiliations:** 1 Chenzhou Third People’s Hospital (Group), Chenzhou, Hunan, China; 2 Affiliated Hospital of Guilin Medical University, Guilin, Guangxi, China

**Keywords:** biomarkers, hematological diseases, metabolomics, sphingolipid metabolism, therapeutic targets

## Abstract

Sphingolipids are not only key structural components of cell membranes but also serve as important signaling molecules, and their metabolic homeostasis is essential for the maintenance of cellular functions. In recent years, with the advancement of technologies such as metabolomics, studies have revealed that sphingolipid metabolic abnormalities are extensively involved in the occurrence and development of various hematological disorders, including immune thrombocytopenia, myeloproliferative neoplasms, graft-versus-host disease, hereditary hematological diseases, and hematologic malignancies. This article aims to provide a systematic review of the fundamental biological functions of sphingolipid metabolism, with a focus on its metabolic reprogramming and the regulatory mechanisms of associated signaling pathways in hematological diseases. By integrating recent advances in both clinical and basic research, this review analyzes potential biomarkers and therapeutic targets within sphingolipid metabolic pathways. Furthermore, it discusses future research directions and the prospects for clinical application in this field, aiming to provide a novel theoretical basis and strategic insights for the precise diagnosis and innovative treatment of hematological diseases.

## 1 Introduction

Sphingolipids are a class of lipid molecules characterized by an amino alcohol backbone, ubiquitously present in cell membranes. They are not only essential components for maintaining membrane structural integrity but also serve as key bioactive molecules involved in various physiological and pathological processes, including cell signaling, proliferation, differentiation, apoptosis, and inflammatory responses (Pokrovsky et al., 2023). In recent years, with the rapid development of high-throughput analytical technologies such as metabolomics and lipidomics, the role of sphingolipid metabolism in various diseases, especially in hematological diseases, has gradually become a research focus (Kumari et al., 2020). Studies have shown that abnormalities in sphingolipid metabolism are closely linked to the determination of cell fate. For instance, ceramide platforms typically induces cell cycle arrest and apoptosis, whereas its phosphorylated derivative, such as sphingosine-1-phosphate (S1P), promotes cell survival and proliferation. The dynamic balance between these two—known as the “sphingolipid rheostat”—is critical for maintaining cellular homeostasis (Hammad et al., 2020; Kono et al., 2022). This disruption of balance, namely the dysregulation of sphingolipid metabolism, has been extensively implicated in the pathological processes of tumorigenesis, immune dysregulation, and various organ diseases (Mallela et al., 2022; Pokrovsky et al., 2023).

In the field of hematological diseases, the metabolic reprogramming of cells, particularly alterations in lipid metabolism, has been confirmed to be closely related to disease pathogenesis, progression, and treatment response (Kumari et al., 2020). The application of metabolomics technologies enables the systematic profiling of metabolic landscapes under disease conditions, thereby revealing the critical role of sphingolipid metabolism. For example, in immune thrombocytopenia (ITP), metabolomic analysis of bone marrow biopsy samples from patients revealed that sphingolipid metabolism and the sphingolipid signaling pathway are among the most significantly enriched pathways for differential metabolites, suggesting a potential association with the underlying pathological mechanisms of ITP and the prediction of responses to different therapeutic agents (Li et al., 2024). Similarly, in metabolomic studies of peripheral blood serum from patients with polycythemia vera (PV), significant alterations in sphingolipid metabolic pathways were observed, and these changes were closely associated with *JAK2* mutations and peripheral blood cell proliferation (Wang et al., 2022). These studies collectively suggest that abnormalities in sphingolipid metabolism are a common feature in hematological diseases.

In addition to its role in myeloproliferative neoplasms and autoimmune hematological diseases, sphingolipid metabolism also plays a significant role in complications associated with hematopoietic stem cell transplantation (HSCT). Allogeneic hematopoietic stem cell transplantation is an effective treatment for various hematologic malignancies, but its efficacy is often limited by graft-versus-host disease (GVHD). Studies have revealed that targeting key enzymes involved in sphingolipid metabolism, such as ceramide platforms synthase 6 (*CerS6*), can modulate the activation and migration of donor T cells via the *N-RAS*/ERK signaling pathway. This approach mitigates both acute and chronic GVHD while preserving the graft-versus-leukemia (GVL) effect, thereby offering a novel strategy to improve transplantation outcomes (Sofi et al., 2022). Furthermore, the metabolic status of patients after transplantation, including alterations in sphingolipid metabolism, is also considered to be associated with the risk of developing GVHD (Kumari et al., 2020). In sickle cell disease (SCD), transfusion of red blood cells stored for different durations leads to age-dependent alterations in sphingolipid metabolism in recipients, which are associated with changes in oxidative stress, inflammatory status, and markers of renal function. This underscores the potential significance of sphingolipid metabolism in transfusion medicine and disease management (Karafin et al., 2025).

Although hematologic diseases are diverse, the ways in which sphingolipid metabolism contributes to their pathogenesis can be summarized around three common features: the balance between ceramide platforms and S1P – ceramide platforms promotes cell death while S1P promotes survival, and the two often change in opposite directions; lysosomal dysfunction – leading to sphingolipid accumulation and subsequent inflammation; and lipid raft remodeling – which alters membrane receptor signaling. The final disease phenotype depends on the affected cell type and the genetic background. This framework helps explain why the same sphingolipid pathway can produce different outcomes in different diseases.

In summary, through its complex metabolic network and signaling functions, sphingolipid metabolism is profoundly involved in core biological processes such as immune regulation, cell proliferation and apoptosis, and inflammatory responses in hematological diseases. In-depth analysis of the molecular mechanisms underlying sphingolipid metabolism abnormalities in different hematological disease contexts not only contributes to clarifying the nature of the diseases but also provides important directions for the development of novel biomarkers and targeted therapeutic strategies. This review will focus on the relationship between sphingolipid metabolism and hematological diseases, systematically review current research progress, and discuss future research and application prospects.

## 2 Sphingolipid metabolism: fundamental biological functions

### 2.1 Structure and classification of sphingolipids

Sphingolipids are structurally divided into three main classes: sphingomyelin, cerebrosides (neutral glycosphingolipids), and gangliosides (acidic glycosphingolipids). All contain a ceramide platforms backbone, and ceramide platforms itself is a central metabolite rather than a separate class (Yang and Chen, 2022). Ceramide platforms, as the central molecule in sphingolipid metabolism, is formed by a sphingoid base linked to a fatty acid chain via an amide bond. The chain length (C16-C24) and degree of saturation of its fatty acid significantly influence its biological activity. For instance, ceramides with different N-acyl chain lengths exhibit distinct phase behavior, interfacial elasticity, and lateral mixing within membranes, thereby modulating the structure and function of membrane microdomains (Fanani and Maggio, 2017). SM, which structurally contains an additional phosphocholine head group, is the most abundant sphingolipid in mammalian cell membranes. Its metabolites, such as S1P, are involved in the regulation of cell proliferation and migration (Yang and Chen, 2022). Glycosphingolipids are formed by adding monosaccharide or polysaccharide chains to ceramide platforms via glycosyltransferases. Simple forms with a single sugar are called cerebrosides (e. g., glucosylceramide platforms, GlcCer), while more complex forms with sialic acid residues are gangliosides (McDonald and Davey, 2021).

The structural diversity of sphingolipids directly dictates their functional specificity. For example, the N-acyl chain length of ceramide influences membrane microdomain formation: symmetric long-chain ceramides tend to form ordered complexes with SM, whereas asymmetric ceramides with very long chains (>C20) may adopt distinct hexagonal arrangements, thereby regulating lipid raft stability (Turpin-Nolan and Brüning, 2020; DiPasquale et al., 2022; Hammerschmidt and Brüning, 2022). The glycan headgroup structure of glycosphingolipids mediates cell recognition and signal transduction. Gangliosides, for instance, enrich within lipid rafts and participate in receptor clustering and immune responses (Takahashi et al., 2022; van der Haar Àvila et al., 2023).

Notably, the function of a given sphingolipid species is highly context-dependent. Taking ceramide as an example, it maintains membrane structural integrity at basal levels but, under stress or at elevated concentrations, induces cytotoxicity by activating mitochondrial pathways or endoplasmic reticulum stress (Park and Park, 2020; Pokrovsky et al., 2023; Wang et al., 2023). Similarly, S1P promotes cell survival and proliferation at low concentrations, whereas its dysregulated signaling drives inflammation and tumor progression (Bassi et al., 2021; Li et al., 2021; Xu et al., 2021). Therefore, the biological effects of sphingolipids are determined not only by their chemical structures but also strictly by their local concentration, subcellular localization, and the specific physiological or pathological context (Pokrovsky et al., 2023; Šakić et al., 2024).

From the perspective of distribution, different sphingolipids exhibit distinct compartmentalization within the cell membrane. The plasma membrane is rich in SM and GIPC, whereas the tonoplast is predominantly composed of glucosylceramide platforms and hydroxylated ceramide platforms (Carmona-Salazar et al., 2021). This differential distribution is closely linked to the pathways of sphingolipid synthesis and transport: after ceramide platforms is synthesized in the endoplasmic reticulum, SM undergoes phosphocholine modification in the Golgi apparatus, while glycosphingolipids require stepwise processing by glycosylation enzyme systems (Liebisch et al., 2020). Dynamic studies have shown that the aggregation state of sphingolipids within membrane microdomains (e. g., lipid rafts) changes in response to external stimuli. For example, under stress conditions, ceramide platforms can form nanodomains ranging from 10 to 200 nm, which recruit pro-apoptotic proteins (Samhan-Arias et al., 2023). This spatial reorganization further amplifies the regulatory potential of sphingolipid molecules in signal transduction.

### 2.2 Sphingolipid metabolic pathways and key enzymes

Sphingolipid metabolism is a highly complex and tightly regulated biochemical network that involves multiple enzymatic pathways, with its core objective being to maintain the dynamic balance among different sphingolipid species. ceramide platforms is widely recognized as the central hub of this metabolic network. It serves not only as the direct precursor for the synthesis of more complex sphingolipids (such as SM and glycosphingolipids), but its own accumulation also directly regulates key biological processes including cell cycle arrest, cell death, and inflammatory responses (Gomez-Larrauri et al., 2021). The generation of ceramide platforms primarily relies on the ceramide platforms synthase (CerS) family, which comprises multiple members. These enzymes utilize fatty acid precursors of varying chain lengths to synthesize ceramide platforms with specific chain lengths, thereby determining the diversity of sphingolipid metabolites (Kim et al., 2021). Studies have indicated that CerS not only functions as a catalytic core but also contains multiple functional domains and is capable of interacting with other proteins within lipid metabolic pathways, which underscores that ceramide platforms and its synthases serve as critical nodes in the sphingolipid metabolic network (Kim et al., 2021). In addition to the synthetic pathways, the catabolism of sphingolipids is equally crucial, involving various hydrolases such as ceramidases and sphingomyelinases, which collectively determine the homeostatic levels of signaling molecules including ceramide platforms and sphingosine.

**Figure 1:**
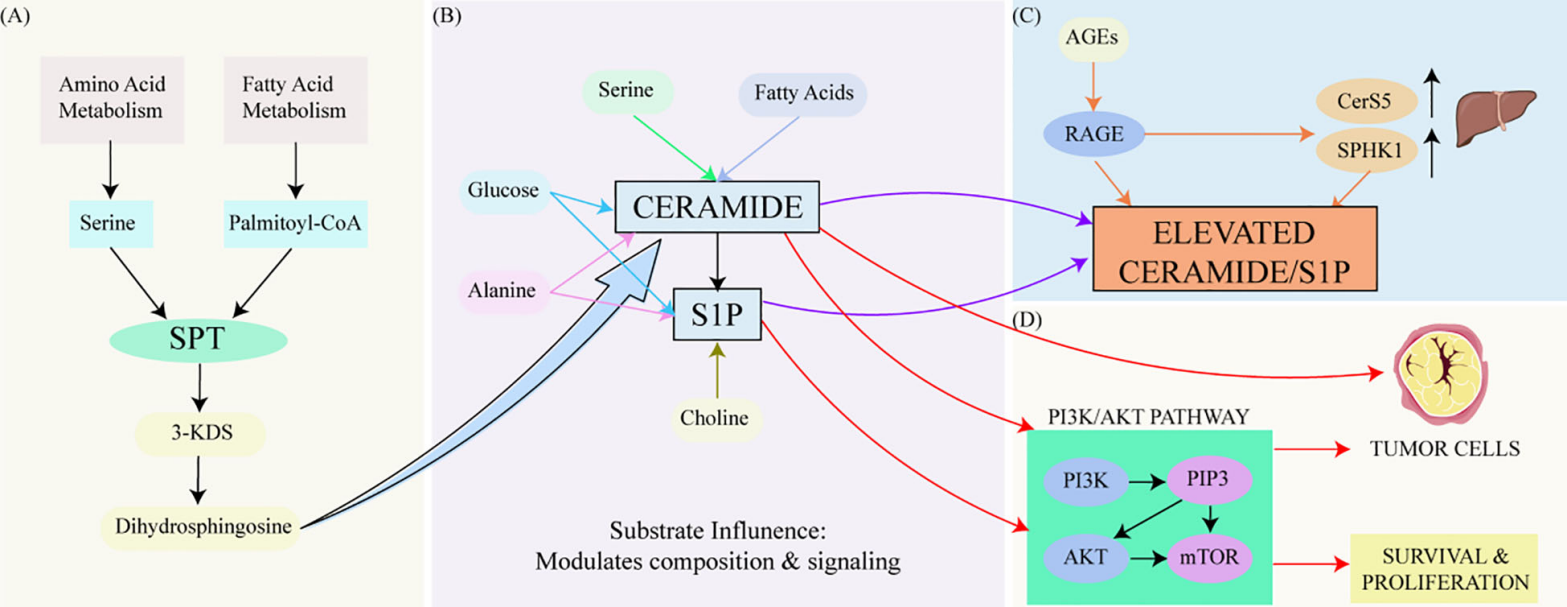
Sphingolipid metabolic network crosstalk and integration in physiological and pathological contexts. **(A)** *De novo* synthesis connection: Initiation of sphingolipid biosynthesis via SPT-mediated condensation of serine (amino acid metabolism-derived) and palmitoyl-CoA (fatty acid metabolism-derived), forming 3-ketodihydrosphingosine (3-KDS) and dihydrosphingosine. This establishes direct cross-talk between amino acid, fatty acid, and sphingolipid pathways (Velazquez et al., 2024). **(B)** Key metabolic intermediates and regulatory inputs: ceramide platforms (ceramide platforms) and S1P levels are dynamically regulated by multiple metabolic substrates. Glucose, amino acids (serine, alanine), choline, and fatty acids of varying chain lengths modulate enzymatic activity and substrate availability, thereby altering sphingolipid composition and signaling functions (Velazquez et al., 2024). **(C)** Pathological crosstalk in insulin resistance: AGEs engage RAGE receptors to upregulate ceramide platforms synthase 5 (*CerS5*) and *SPHK1*, driving elevated ceramide platforms/S1P production that exacerbates metabolic dysfunction (Mastrocola et al., 2021). **(D)** Pathological crosstalk in cancer: Oncogenic signaling (e. g., PI3K/AKT pathway) cooperates with dysregulated sphingolipid metabolism in tumor cells, promoting survival and proliferation (Gulhane and Singh, 2022). PI3K/AKT/mTOR pathway: Core oncogenic cascade (PI3K→PIP3→AKT→mTOR) that interfaces with sphingolipid remodeling in cancer (Gulhane and Singh, 2022). This schematic incorporates the following third-party icons, reproduced without modification: the “Healthy_liver” icon by Jan-Clusmann (CC0 https://creativecommons.org/publicdomain/zero/1.0/); and the “Tumor” icon by Servier (CC-BY 3.0 Unported https://creativecommons.org/licenses/by/3.0/).

Sphingolipid metabolic pathways do not exist in isolation but rather extensively intersect with other lipid and energy metabolic pathways, constituting a complex metabolic network. This crosstalk is manifested at multiple levels (**Figure 1**). First, the *de novo* synthesis of sphingolipids begins with the condensation of serine and palmitoyl-CoA catalyzed by serine palmitoyltransferase (SPT), which directly connects amino acid metabolism and fatty acid metabolism (Velazquez et al., 2024). Second, the levels of key metabolic intermediates such as ceramide platforms and S1P are regulated by inputs from various metabolic pathways. These findings indicate that the sphingolipid metabolic network serves as a crucial hub for cells to sense and integrate metabolic changes from both internal and external environments. The maintenance of its homeostasis is essential for cellular functions, whereas its dysregulation is closely associated with various diseases.

### 2.3 The role of sphingolipids in cell signal transduction

**Figure 2:**
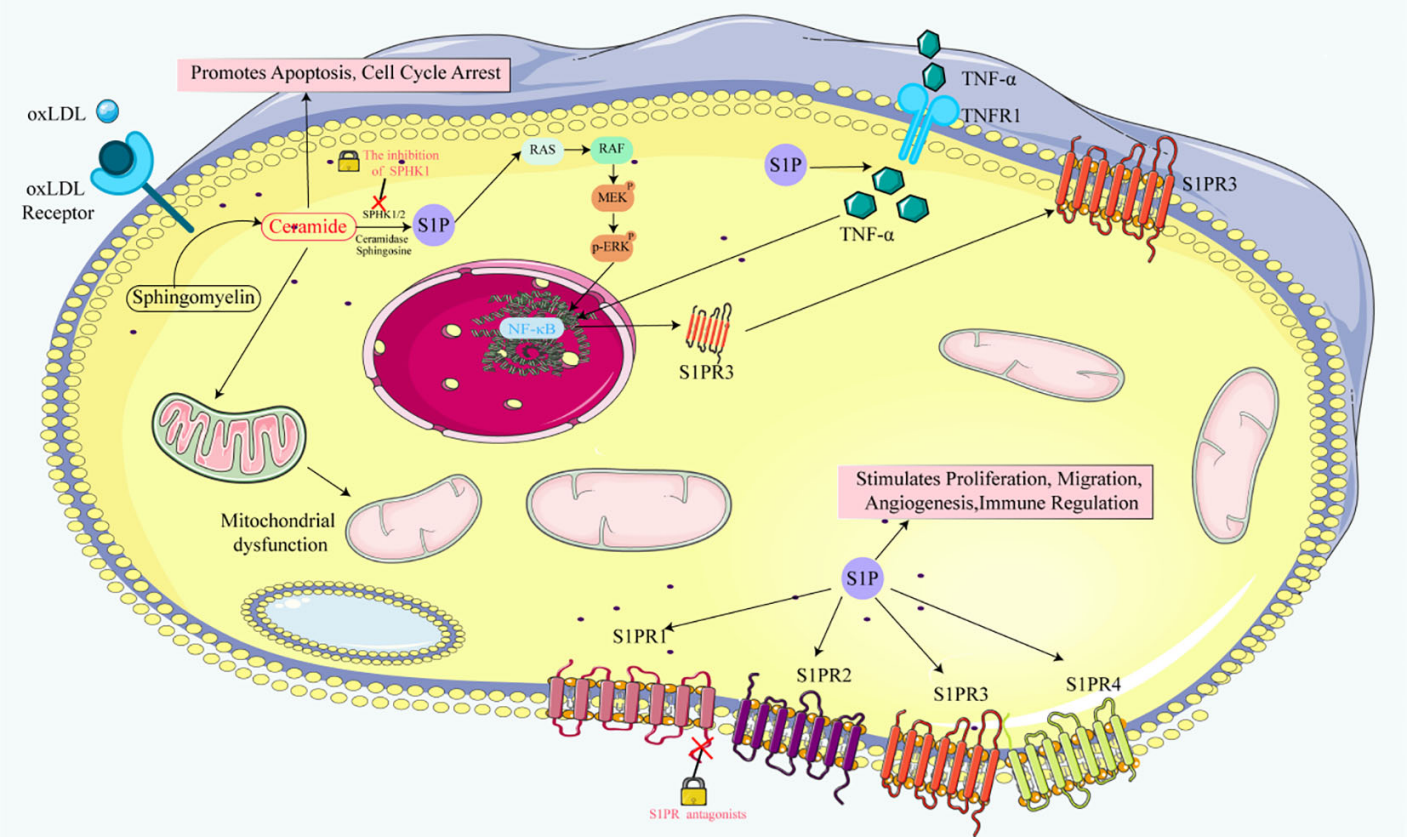
Schematic representation of the sphingolipid metabolic pathway and its role in cellular signaling transduction in mammalian cells. This schematic incorporates icons from Servier Medical Art (“Emptycell-3d-1”, “mitochondrium-6”, “7helix-receptor-membrane”, “mitochondrium-3”; CC-BY 3.0 Unported https://creativecommons.org/licenses/by/3.0/) and from Helicase 11 (“Simple_receptor_2”, “Simple_receptor_4”; CC-BY SA 4.0 https://creativecommons.org/licenses/by-sa/4.0/). All icons were adapted with modifications.

Sphingolipids and their metabolites function as bioactive molecules and play a crucial role in cell signal transduction (**Figure 2**). In atherosclerosis, oxidized low-density lipoprotein (oxLDL) can activate the SM/ceramide platforms pathway, leading to vascular cell proliferation, apoptosis, and inflammation (Augé et al., 2000). Furthermore, the accumulation of ceramide platforms can induce mitochondrial dysfunction and apoptosis, a mechanism that holds significant implications in various diseases such as cancer and neurodegenerative disorders (Fugio et al., 2020). Beyond their role in immune signaling, sphingolipids also interact with core metabolic and oncogenic pathways. For example, the availability of glucose, specific amino acids (such as serine and alanine), choline, and fatty acids of varying chain lengths can reshape the composition and signaling functions of sphingolipids by influencing the activity of relevant enzymes or by being directly incorporated as substrates (Velazquez et al., 2024). Under pathological conditions, the interplay within this metabolic network becomes even more pronounced. For example, in an insulin resistance model, the accumulation of advanced glycation end products (AGEs) alters the expression of key enzymes such as ceramide platforms synthase 5 (*CerS5*) and sphingosine kinase 1 (*SPHK1*) in the liver via their receptor (RAGE), leading to abnormally elevated levels of ceramide platforms and S1P, thereby contributing to disease pathogenesis (Mastrocola et al., 2021). Furthermore, in tumor cells, the reprogramming of sphingolipid metabolism is closely intertwined with classical signaling pathways such as PI3K/AKT, synergistically driving the growth and survival of cancer cells (Gulhane and Singh, 2022).

Sphingolipid metabolites also influence immune cell function and inflammatory responses by modulating key signaling pathways such as *N-RAS*/ERK. In acute myeloid leukemia (AML), S1PR3 has been identified as a downstream signaling molecule of the TNFα-NF-κB axis, regulating the differentiation of hematopoietic stem cells and leukemic stem cells by activating inflammatory programs (Yang et al., 2021). Similarly, in immune cells, S1P promotes lymphocyte migration in an *S1PR1*-dependent manner, influencing the spatiotemporal distribution of immune responses (Sasset et al., 2023). Furthermore, ceramide platforms can promote the release of inflammatory cytokines (such as tumor necrosis factor-alpha, TNF-α) and is involved in the pathological processes of autoimmune diseases like multiple sclerosis (Jana and Pahan, 2010).

The complexity of sphingolipid signaling pathways is also reflected in the compartmentalized nature of their metabolism. The distribution and function of sphingolipids vary across different subcellular structures. For example, ceramide platforms in mitochondria is directly involved in initiating apoptotic signals, whereas S1P at the plasma membrane regulates cell migration through receptor-mediated signal transduction (Canals and Clarke, 2022). This compartmentalized regulation provides a theoretical basis for therapeutic strategies targeting sphingolipid metabolism. For instance, inhibiting *SPHK1* or using S1PR antagonists can modulate sphingolipid homeostasis, demonstrating therapeutic potential in cancer and autoimmune diseases (Noujarède et al., 2023). Furthermore, research on sphingolipid metabolism in plants has also provided insights for animal models, such as the mechanism of action of long-chain bases (LCBs) in programmed cell death (Briegas et al., 2025).

**Figure 3:**
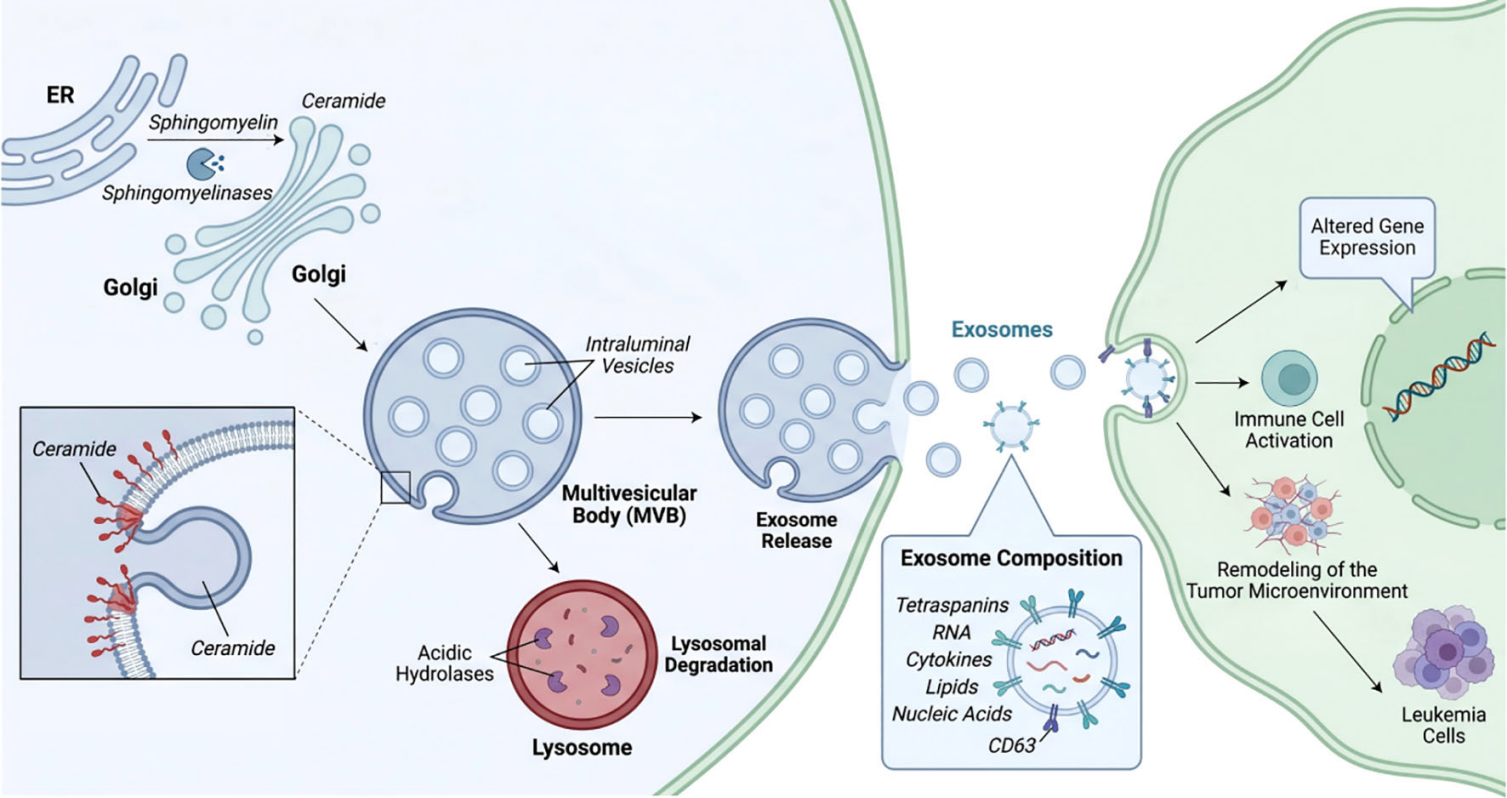
The role of sphingolipids (particularly ceramides) in regulating exosome biogenesis.

Beyond their well-established roles in regulating classical cell fate decisions, sphingolipids and their metabolites, particularly ceramide, play a pivotal role in modulating subcellular organelle function and intercellular communication (**Figure 3**). Recent studies have revealed a central function for ceramide in regulating lysosomal integrity, multivesicular body (MVB) maturation, and exosome release. In various cell types, the accumulation of ceramide has been shown to influence lysosomal integrity and function (Yuyama et al., 2020). For instance, ceramide can directly interact with the lysosomal membrane, altering its permeability, or it can affect its degradative capacity by modulating the activity of lysosome-associated membrane proteins and enzymes (Milliken et al., 2020). This regulation is crucial for maintaining cellular homeostasis, and its dysregulation is linked to a variety of disease states.

More importantly, ceramide is a key lipid molecule driving the biogenesis and release of exosomes (Matsui et al., 2021). Exosomes originate from intraluminal vesicles formed by the inward budding of the limiting membrane of late endosomes, known as MVBs (Marie et al., 2023). Studies have shown that the local generation of ceramide, due to its inherent cone-shaped molecular structure, can promote the inward budding of the MVB membrane, directly participating in the formation of intraluminal vesicles. This process is independent of the classical ESCRT (endosomal sorting complexes required for transport) machinery and constitutes a major pathway for exosome generation (Yang et al., 2024). Consequently, the homeostasis of sphingolipid metabolism directly influences the yield, lipid composition, and cargo (e. g., proteins, nucleic acids) of exosomes (Horbay et al., 2022).

The interaction between lysosomes and MVBs acts as a “switch” that determines the fate of exosomes. MVBs can either fuse with lysosomes for degradation of their contents or with the plasma membrane to release their intraluminal vesicles as exosomes. Sphingolipid metabolism, particularly the dynamic balance between ceramide and S1P, has been shown to regulate this decision-making process (Rincón-Riveros et al., 2021). For example, S1P signaling may favor MVB exocytosis, while the accumulation of specific ceramide species may route MVBs toward the lysosomal degradation pathway (Riese et al., 2026). In hematologic disorders, aberrant sphingolipid metabolism in tumor or immune cells may, through the mechanisms described above, lead to abnormal release of exosomes with specific immunomodulatory or pro-survival functions, thereby remodeling the tumor microenvironment or exacerbating immune dysregulation (Raza et al., 2022). For instance, in chronic lymphocytic leukemia and diffuse large B-cell lymphoma, tumor cells may undergo sphingolipid metabolic reprogramming to increase the production of pro-survival S1P and decrease pro-apoptotic ceramide, potentially accompanied by increased secretion of pro-tumor exosomes. These exosomes could suppress effector T cell function and recruit immunosuppressive cells, establishing an immunosuppressive niche (Talbot et al., 2020). This area of research tightly links fundamental cell biology with disease pathophysiology and represents a critical direction for future translational studies.

In summary, sphingolipids and their metabolites regulate cell fate through a multi-layered signaling network, with their mechanisms of action involving the intricate regulation of metabolic enzymes, receptors, and downstream effector molecules. Future research should further elucidate the spatiotemporal dynamics of sphingolipid metabolism and its disease-specific targets, providing a rationale for the development of novel therapeutic strategies.

This figure summarizes the key components and mechanisms of sphingolipid-mediated signal transduction. The pathway is initiated by extracellular stimuli such as oxidized oxLDL, which binds to its receptor (e. g., oxLDL Receptor) and activates sphingomyelinase to generate ceramide platforms (Augé et al., 2000). ceramide platforms acts as a central bioactive lipid, promoting mitochondrial dysfunction and apoptosis, processes implicated in diseases like atherosclerosis, cancer, and neurodegeneration. ceramide platforms can be further metabolized to sphingosine-1-phosphate (S1P), which signals through specific G protein-coupled receptors (e. g., *S1PR1*, *S1PR3*) to regulate diverse cellular responses including cell proliferation, differentiation, immune cell migration, and inflammatory gene expression via pathways such as *N-RAS*/ERK and TNFα-NF-κB (Yang et al., 2021; Sasset et al., 2023). The illustration highlights the compartmentalization of sphingolipid signaling, with ceramide platforms exerting pro-apoptotic effects primarily at the mitochondria, while S1P operates at the plasma membrane to influence cell motility and immune responses. Potential therapeutic targets, including the inhibition of *SPHK1* or S1P receptor antagonism, are indicated, underscoring the relevance of modulating sphingolipid metabolism in treating autoimmune diseases and cancer (Canals and Clarke, 2022; Noujarède et al., 2023).

As depicted, the intracellular generation of ceramide directly promotes the formation of intraluminal vesicles within MVBs. The fate of MVBs is regulated by factors including sphingolipid metabolic balance; they can be either trafficked to lysosomes for degradation or fuse with the plasma membrane to release exosomes. These released exosomes act as intercellular messengers, delivering their cargo of proteins, lipids, and nucleic acids to recipient cells, thereby mediating long-range signaling, immune modulation, and tumor microenvironment remodeling in (hematologic) diseases.

## 3 Sphingolipid metabolism abnormalities and immune thrombocytopenia

### 3.1 Pathological mechanisms and metabolic characteristics of ITP

**Figure 4:**
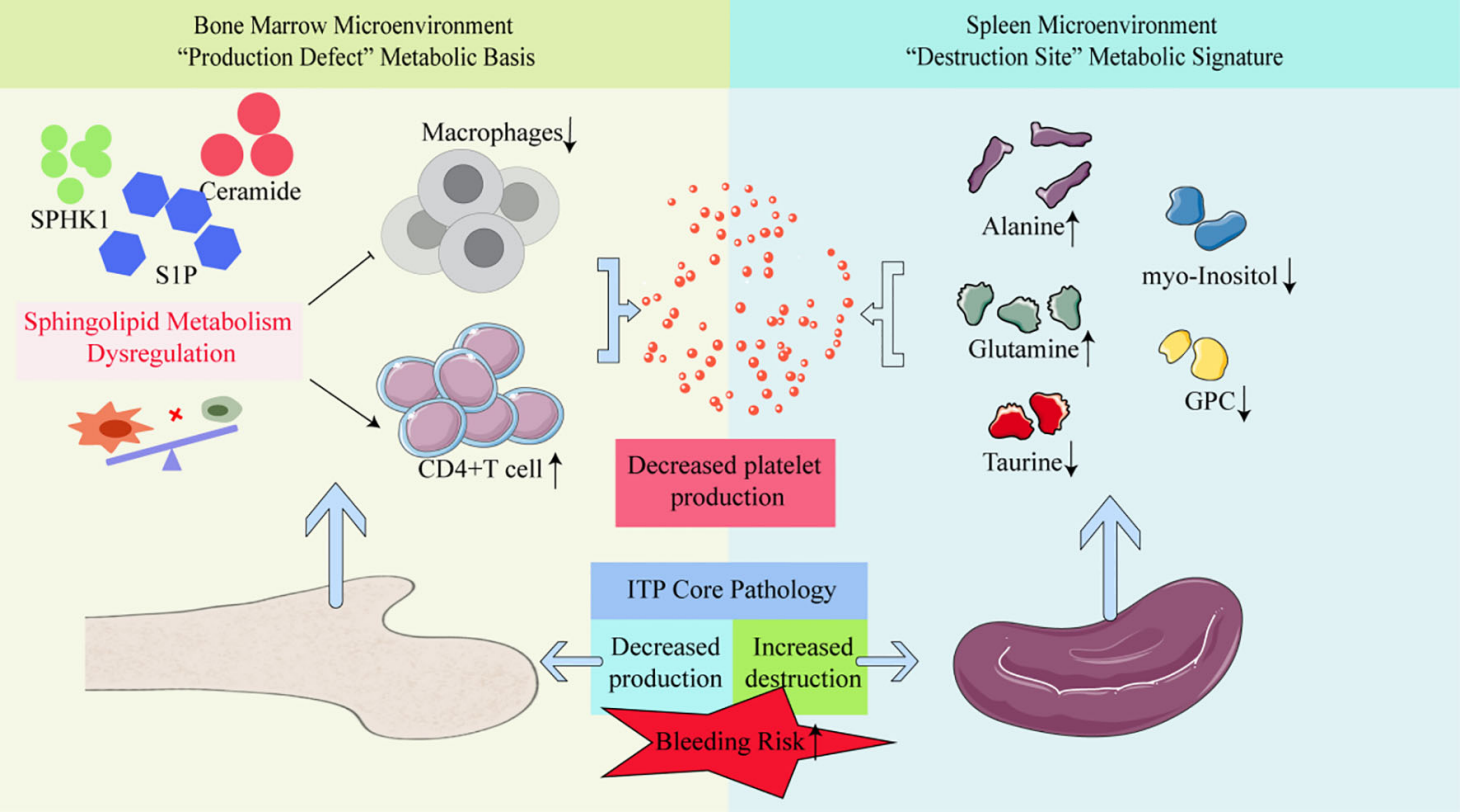
Systemic metabolic dysregulation in ITP. This schematic incorporates icons from Servier Medical Art (“Spleen-1”, “lymphocytes-3”; CC-BY 3.0 Unported https://creativecommons.org/licenses/by/3.0/) and from El-Jayawant (“Bone_marrow”; CC-BY 4.0 Unported https://creativecommons.org/licenses/by/4.0/). All icons were reproduced exactly without modification.

ITP is an autoimmune disease. Traditionally, its pathological mechanisms have focused on immune cell abnormalities and autoantibody-mediated platelet destruction. However, recent metabolomics studies have revealed profound metabolic reprogramming. A prospective analysis of bone marrow samples from 91 ITP patients quantified 4,494 metabolites and identified 876 differential metabolites and 181 distinct pathways between newly diagnosed and chronic patients, among which the sphingolipid metabolism pathway was particularly significantly altered. As key bioactive lipids, the abnormal activity of sphingolipids may exacerbate impaired platelet production and destruction by influencing megakaryocyte maturation, immune cell activity, and inflammatory responses. Furthermore, other lipid metabolic pathways, such as glycerophospholipid metabolism, are also associated with treatment response, underscoring the central role of the lipid metabolic network in the pathophysiology of ITP (Li et al., 2024) (**Figure 4**). However, this single-center study lacked independent validation and did not correct for multiple comparisons. Prior treatments and disease duration were not adjusted. So the observed sphingolipid alterations are associations, not causal. Future studies need treatment-naïve patients, multi-center replication, and functional experiments.

In addition to the bone marrow, the spleen, as the primary site of platelet destruction, also exhibits significant metabolic dysregulation in ITP. A metabolomics study based on nuclear magnetic resonance spectroscopy found that the concentrations of various amino acids (such as alanine and glutamine) were increased in the spleen tissue of ITP patients, while the levels of metabolites such as taurine and inositol were significantly decreased. Among these, the decrease in taurine level was the most significant metabolic marker distinguishing ITP from normal spleen, demonstrating high sensitivity and specificity (Wen et al., 2022). Taurine is not a direct sphingolipid, but it can modulate ceramide synthesis by influencing serine palmitoyltransferase (SPT) activity, thereby linking this finding to sphingolipid metabolism (Huang et al., 2026). More importantly, the taurine level in the spleen was negatively correlated with the efficacy of splenectomy, suggesting its potential as a predictive biomarker for surgical response (Wen et al., 2022).

This schematic illustrates the compartmentalized metabolic alterations in ITP, highlighting the bone marrow microenvironment associated with platelet production defects and the splenic microenvironment responsible for platelet destruction. In the bone marrow, dysregulated sphingolipid metabolism (e. g., ceramide platforms and S1P) promotes pro-inflammatory polarization of CD4+ T cells and impairs megakaryocyte maturation, contributing to decreased platelet production. Metabolomic analysis identified 876 significantly dysregulated metabolites, with the sphingolipid pathway being the most altered (p<0. 001) (Li et al., 2024). In the spleen, taurine deficiency emerges as a key metabolic signature, inversely correlating with splenectomy response (r = -0. 52, p<0. 003); additional alterations include elevated alanine and glutamine and reduced myo-inositol and glycerophosphocholine (GPC) (Wen et al., 2022). Together, these organ-specific metabolic disruptions drive ITP pathology by simultaneously reducing platelet production and increasing destruction.

Taken together, from the bone marrow to the spleen, ITP exhibits systemic metabolic dysregulation, among which abnormalities in the sphingolipid metabolic pathway are a core feature of the bone marrow microenvironment, while specific metabolite alterations in the spleen are closely associated with disease diagnosis and treatment prognosis, thereby forming a complex metabolic pathological network in ITP.

### 3.2 Sphingolipid metabolism-related biomarkers and prediction of treatment response

Based on patient responses to five different treatment regimens, researchers utilized machine learning algorithms (such as the Boruta algorithm and random forest algorithm) to screen and evaluate the importance of metabolites. The findings revealed that lipids and their metabolites—including long-chain fatty acids, oxidized lipids, glycerophospholipids, and biosynthetic products of phosphatidylcholine (PC) and phosphatidylethanolamine—have helped distinguish treatment responses to different drugs (Li et al., 2024). This indicates that analyzing the sphingolipid metabolic profile within the bone marrow microenvironment holds promise for the discovery of biomarkers predictive of individualized treatment responses in ITP patients.

However, despite these promising findings, their clinical translational value must be interpreted with caution. First, reproducibility is a critical concern. The biomarker profiles reported across different studies show marked discrepancies, which may stem from heterogeneity in sample sources (e.g., bone marrow vs. peripheral blood), detection methods (e.g., different liquid chromatography-tandem mass spectrometry platforms), and downstream data analysis workflows. Second, most of these studies are cross-sectional, revealing strong correlations but rarely establishing causation. Therefore, it remains to be determined whether certain metabolite changes are drivers of the disease or merely epiphenomena of the disease state. Furthermore, although mass spectrometry-based sphingolipidomics (e.g., LC-MS/MS) can simultaneously detect dozens of sphingolipid species with high sensitivity and specificity (e.g., linear range r² > 0.99, recovery 80%–120%), the technology itself still faces challenges. For instance, the discrimination of different lipid isomers, standardization of absolute quantification, and batch effects in high-throughput analyses may all affect the accuracy of results and cross-study comparability. Consequently, for these findings to progress from “candidate biomarkers” to mature diagnostic tools for clinical use, they require multi-center, prospective validation in independent cohorts to assess their robustness across different populations and disease subtypes.

## 4 The role of sphingolipid metabolism in myeloproliferative neoplasms and hematologic malignancies

### 4.1 Metabolic alterations in multiple myeloma

**Figure 5:**
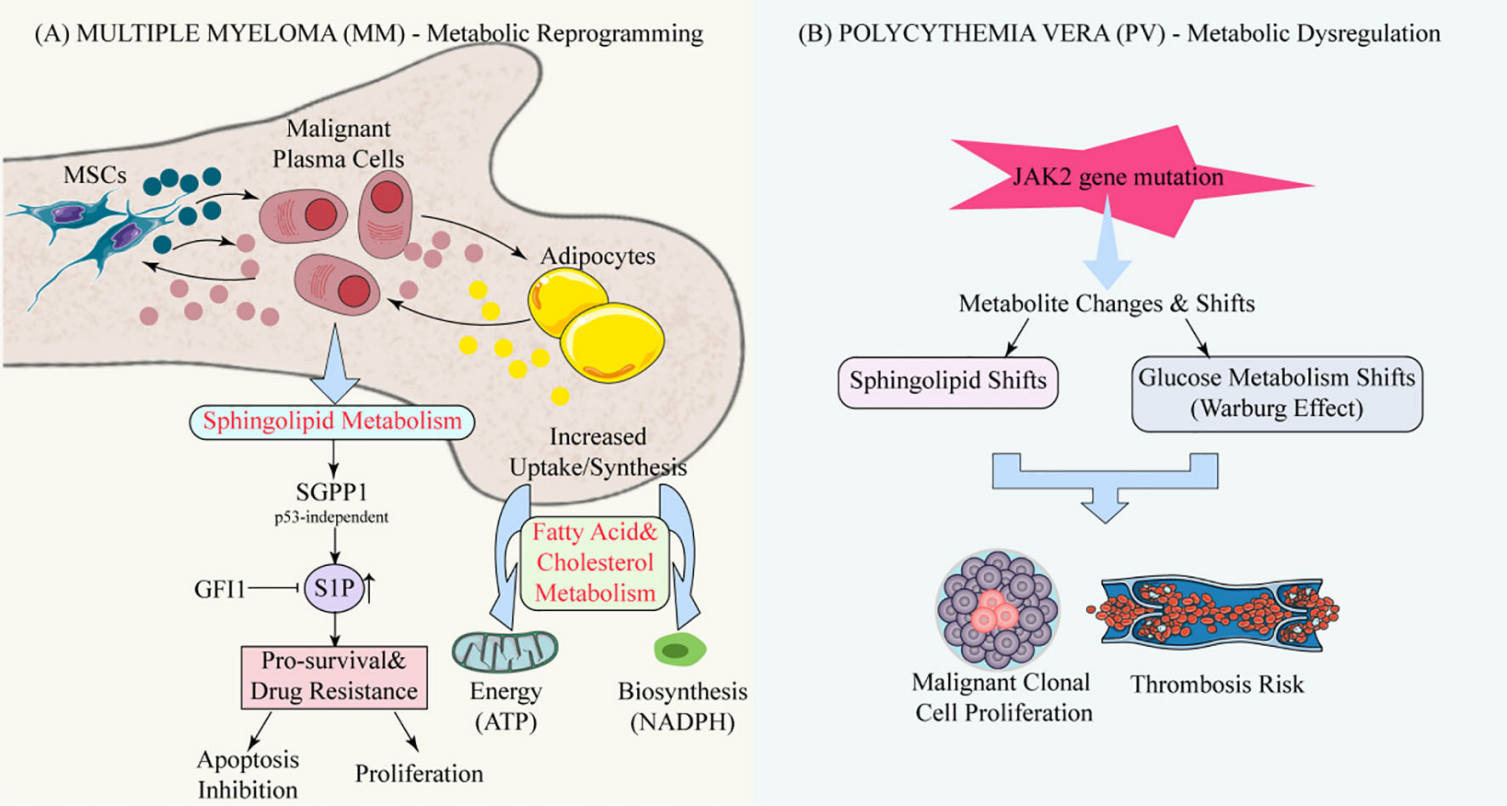
Comparative metabolic dysregulation in myeloproliferative neoplasms. This schematic illustrates the distinct yet convergent metabolic reprogramming in MM and PV, highlighting the central role of sphingolipid metabolism. In the MM module **(A)** the bone marrow microenvironment—comprising malignant plasma cells, mesenchymal stromal cells (MSCs), and adipocytes—exhibits dysregulated sphingolipid metabolism. Key alterations include GFI1-mediated suppression of SGPP1, leading to accumulation of S1P, which promotes tumor cell survival via p53-independent mechanisms (Petrusca et al., 2022b). Concurrent perturbations in energy metabolism (e. g., ATP production) support MM progression. In the PV module **(B)** driven by JAK2 mutation, metabolic dysregulation involves aberrant sphingolipid, fatty acid, and amino acid pathways, fostering erythroid proliferation (Wang et al., 2022). This schematic incorporates icons from El-Jayawant (“Bone_marrow”, “plasma_cell”; CC-BY 4.0 Unported https://creativecommons.org/licenses/by/4.0/), Xi-Chen (“Morula_embryo”; CC0 https://creativecommons.org/publicdomain/zero/1.0/), and Servier Medical Art (“Adipocyte-2”, “mitochondrium-blue”, “venous-thrombosis-4”; CC-BY 3.0 Unported https://creativecommons.org/licenses/by/3.0/). All icons were reproduced exactly without modification.

Metabolic reprogramming is a hallmark of cancer and plays a critical role in the pathogenesis, progression, and therapeutic resistance of hematological diseases such as multiple myeloma (MM) and PV (Weir et al., 2023) (**Figure 5**). In MM, malignant plasma cells support their growth, survival, and adaptation to the tumor microenvironment by altering multiple metabolic pathways, including sphingolipid metabolism, fatty acid metabolism, and cholesterol metabolism (Torcasio et al., 2023). These metabolic alterations not only provide energy and biosynthetic precursors for tumor cells but also profoundly shape disease progression by influencing cell signaling, immune responses, and drug sensitivity (Petrusca et al., 2022a). For example, growth factor independence 1 (*GFI1*) regulates sphingolipid metabolism by suppressing the expression of *SGPP1*, thereby increasing S1P levels and supporting MM cell survival in a p53-independent manner (Petrusca et al., 2022b). Furthermore, the interaction between MM cells and the bone marrow microenvironment (such as mesenchymal stromal cells and adipocytes) further induces metabolic reprogramming, forming a feed-forward loop that supports tumor growth (Fairfield et al., 2020). Metabolic abnormalities are also associated with the prognosis of MM, and the overexpression of energy metabolism-related genes correlates with poorer progression-free survival and overall survival in patients (Evans et al., 2022).

### 4.2 Metabolic alterations in polycythemia vera

Similarly, in PV, metabolic abnormalities are closely associated with disease pathogenesis and cell proliferation. *JAK2* gene mutation is one of the primary causes of peripheral blood cell proliferation in PV, and metabolomic analyses have revealed significant metabolic alterations in the serum of PV patients (Wang et al., 2022). Compared with healthy controls, 33 endogenous metabolites were significantly altered in PV patients, involving multiple pathways including fatty acid metabolism, glucose metabolism, sphingolipid metabolism, and amino acid metabolism (Wang et al., 2022). Among these, seven metabolites were identified by statistical correlation analysis as being linked to JAK2 mutations. The claim that two of them may promote peripheral blood cell proliferation is based on these correlations, not on direct experimental evidence (Wang et al., 2022). These findings suggest that metabolic abnormalities are a novel feature of malignant clonal cell proliferation in PV. Furthermore, the metabolic profile of PV patients is fundamentally different from that of patients with secondary erythrocytosis. For instance, in PV patients, metabolites such as thyrotropin-releasing hormone and 3-sulfinoalanine are upregulated, whereas 4-hydroxyretinoic acid and deoxyuridine are downregulated. These differences may provide potential biomarkers for differential diagnosis (Yıldırım et al., 2025). Lipid metabolism disorders, particularly decreased levels of high-density lipoprotein cholesterol (HDL-C), are also associated with an increased risk of thrombosis in PV patients (Lai et al., 2023).

However, this serum metabolomics study was single-center and lacked independent validation. The seven metabolites linked to JAK2 mutations were identified by correlation analysis, not functional experiments – so they may be bystanders rather than drivers of cell proliferation. Whether the two metabolites actually promote peripheral blood cell expansion remains unproven. The differential metabolites for PV versus secondary erythrocytosis also need replication in larger, multi-center cohorts. The HDL-C finding is observational; causality and clinical utility await prospective studies.

In summary, the metabolic alterations in MM and PV, especially the dysregulation of sphingolipid metabolism, may be key factors driving malignant clonal cell proliferation and disease progression, providing an important perspective for understanding disease mechanisms and developing new therapeutic strategies.

### 4.3 Dysregulation of ceramide platforms metabolic pathways and tumorigenesis

**Figure 6:**
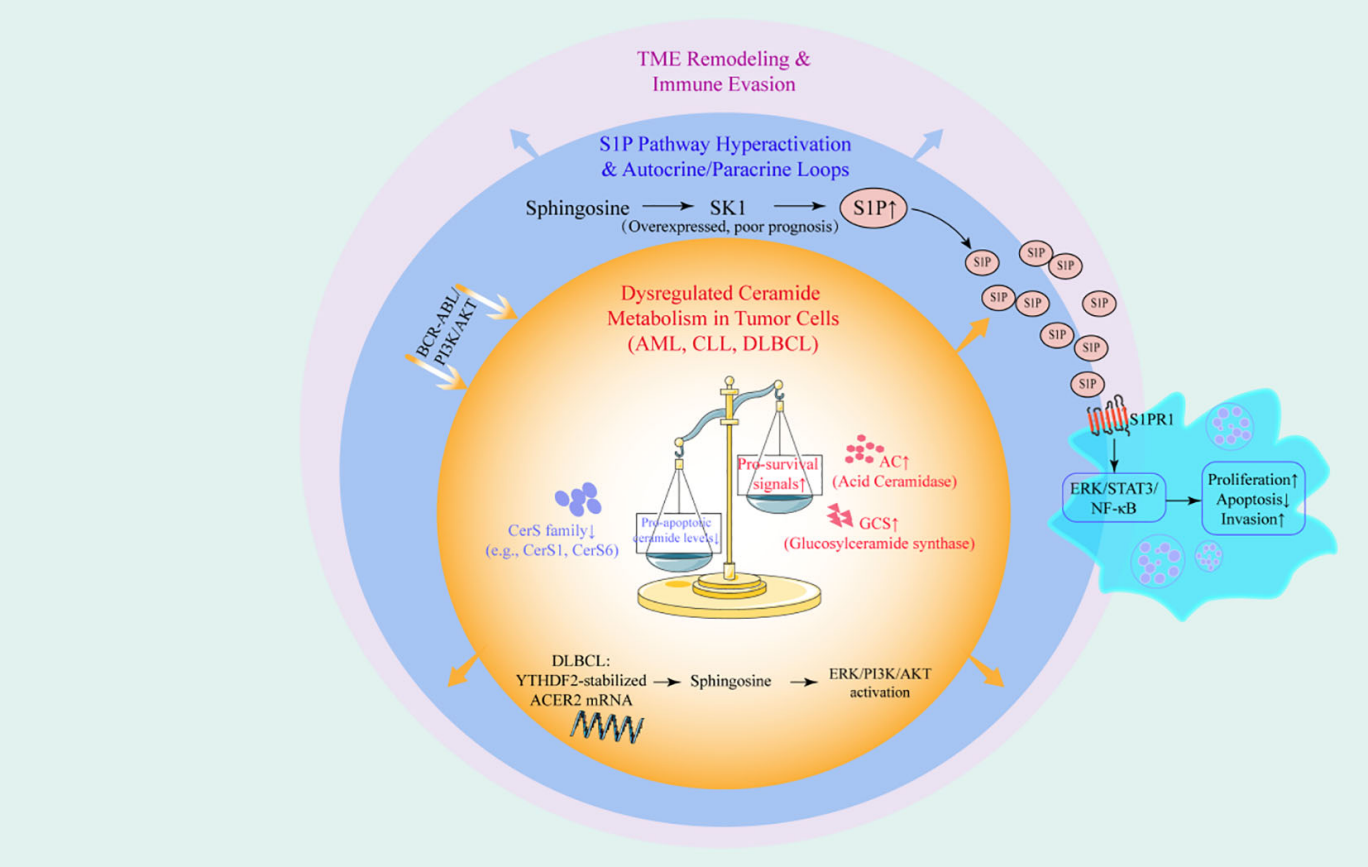
Sphingolipid metabolic reprogramming in hematological malignancies: from intracellular ceramide platforms-S1P imbalance to immunosuppressive niche formation.

Dysregulation of ceramide platforms metabolic pathways plays a critical role in the occurrence and development of various hematologic malignancies (**Figure 6**). In AML and chronic lymphocytic leukemia (CLL), the downregulation or suppressed activity of *de novo* ceramide platforms synthases (such as the CerS family) has been widely reported (Raza et al., 2022). This downregulation leads to a reduction in pro-apoptotic signaling within the cell, thereby conferring a survival advantage on tumor cells. For instance, studies have shown that the loss of *CerS6* can significantly ameliorate GVHD by inhibiting T cell receptor signaling and ERK activation, while concurrently preserving the anti-leukemic effect (Sofi et al., 2022). Furthermore, tumor cells also reduce ceramide platforms levels by upregulating ceramide platforms-degrading enzymes (such as acid ceramidase) or glycosylating enzymes (glucosylceramide platforms synthase) (Chen et al., 2024). For example, in diffuse large B-cell lymphoma (DLBCL), *YTHDF2* stabilizes alkaline ceramidase 2 (*ACER2*) mRNA in an m6A-dependent manner, promoting the hydrolysis of ceramide platforms to sphingosine. This, in turn, activates the ERK and PI3K/AKT pathways, driving tumor progression (Chen et al., 2024). Oncogenic signaling pathways such as PI3K/AKT and *BCR-ABL* can directly regulate the expression and activity of enzymes involved in ceramide platforms metabolism. In chronic myeloid leukemia (CML), *BCR-ABL* activates *SPHK1* via the PI3K/AKT pathway, converting pro-apoptotic ceramide platforms into pro-survival S1P, thereby promoting drug resistance in leukemia cells (Gao et al., 2022). These findings reveal the significant potential of the ceramide platforms metabolic pathway as a therapeutic target in hematologic malignancies.

This schematic illustrates the tripartite dysregulation of sphingolipid metabolism that drives progression and immune evasion in blood cancers. 1) Intracellular ceramide platforms Metabolic Dysregulation: Within the tumor cell (representing AML, CLL, DLBCL, etc.), a metabolic imbalance is depicted where *de novo* ceramide platforms synthesis (e. g., via CerS enzymes) is suppressed, while ceramide platforms degradation pathways (e. g., via acid ceramidase, AC) are upregulated. This shift reduces pro-apoptotic ceramide platforms levels and generates sphingosine (Raza et al., 2022; Chen et al., 2024). 2) Aberrant S1P Pathway Activation & Autocrine/Paracrine Loop: Sphingosine is phosphorylated by overexpressed sphingosine kinase 1 (SK1) to produce S1P. S1P is secreted and acts in an autocrine/paracrine manner by binding to S1P receptors (e. g., *S1PR1*) on the tumor cell surface, activating downstream pro-survival and proliferative pathways (e. g., ERK, PI3K/AKT, STAT3) (Petrusca et al., 2022a; Li et al., 2024; Xu et al., 2025).

## 5 Sphingolipid metabolism and graft-versus-host disease

### 5.1 Immunometabolic characteristics of GVHD

**Figure 7:**
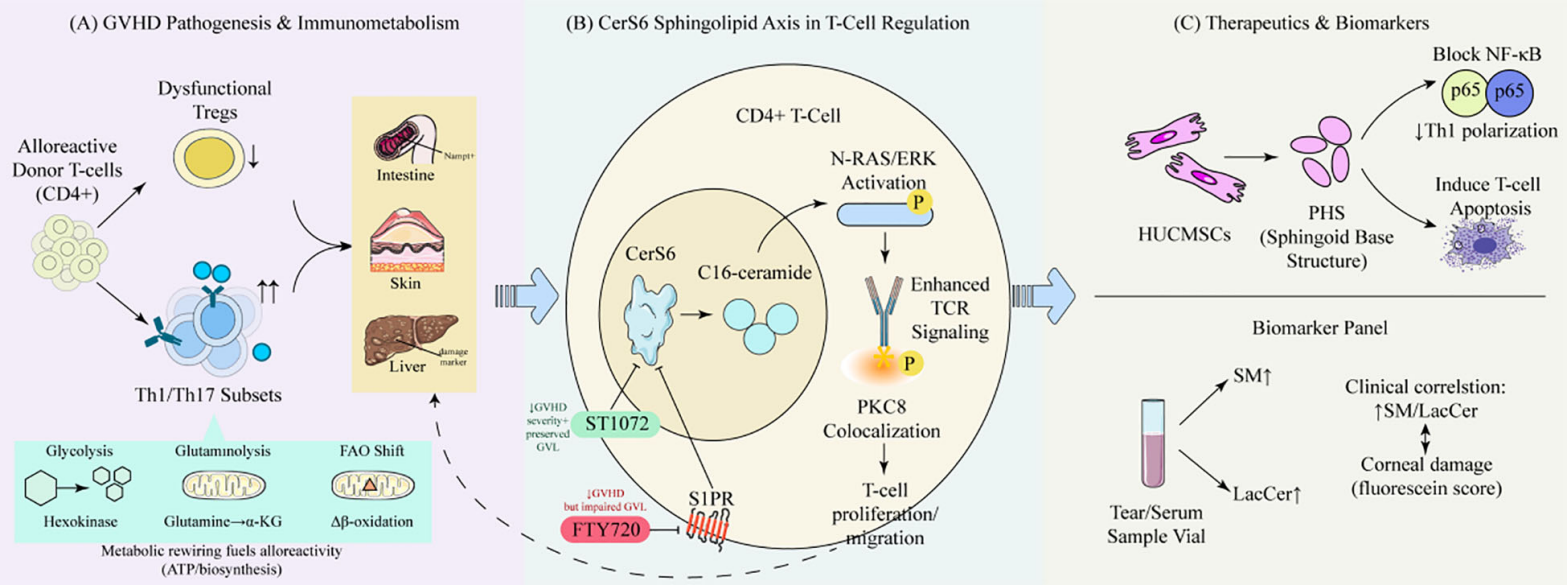
Targeting the *CerS6*-sphingolipid axis in graft-versus-host disease: mechanisms and therapeutic implications. This schematic illustrates the central role of sphingolipid metabolism, specifically mediated by *CerS6*, in the immunopathogenesis of GVHD and its potential as a therapeutic target. The panel **(A)** (GVHD Pathogenesis & Immunometabolism) depicts donor T-cell activation and differentiation into pathogenic Th1/Th17 subsets within the alloreactive microenvironment, characterized by metabolic reprogramming toward glycolysis and glutaminolysis (Mhandire et al., 2021). The panel **(B)** (*CerS6* Sphingolipid Axis) highlights how *CerS6*-generated ceramide platforms activate the *N-RAS*/ERK signaling pathway, enhancing TCR-mediated activation and PKCθ co-localization in CD4+ T cells, thereby promoting their migration to GVHD target organs (Brown and Byersdorfer, 2020; Sofi et al., 2022). Pharmacological inhibition of *CerS6* with ST1072 selectively suppresses T-cell activation and trafficking while preserving graft-versus-leukemia (GVL) effects (Sofi et al., 2022). The panel **(C)** (Therapeutics & Biomarkers) summarizes additional therapeutic strategies, including the upregulation of phytosphingosine (PHS) by HUCMSCs to inhibit pro-inflammatory T-cell responses via NF-κB suppression (Hong et al., 2023), and the identification of sphingolipid metabolites (e. g., SMs and lactosylceramide platforms) as biomarkers correlating with clinical manifestations in ocular chronic GVHD (Ma et al., 2021). This schematic incorporates icons from multiple sources: “Cirrhotic_liver” by Jan-Clusmann (CC0 https://creativecommons.org/publicdomain/zero/1.0/); “Skin-second-degree-burn”, “mitochondrium-yellow”, and “small-intestine-crosssection” by Servier Medical Art (CC-BY 3.0 Unported https://creativecommons.org/licenses/by/3.0/); “B-cell_cluster” by El-Jayawant (CC-BY 4.0 Unported https://creativecommons.org/licenses/by/4.0/, adapted with modifications); and “Mast_cell_degranulation_1” by Sebastian-Reinig (CC-BY 4.0 Unported https://creativecommons.org/licenses/by/4.0/). All other icons were reproduced exactly without modification.

The occurrence of GVHD is closely associated with the metabolic reprogramming of immune cells, particularly the enhanced dependence of donor T cells on sphingolipid metabolism (**Figure 7**). Studies have shown that during the development of GVHD, donor CD4+ T cells differentiate into pathogenic Th1 and Th17 subsets, while the function of immunosuppressive regulatory T cells (Tregs) is impaired (Mhandire et al., 2021). This immunometabolic reprogramming is characterized by the activation of glycolysis and glutaminolysis, accompanied by alterations in fatty acid oxidation metabolism, thereby providing the energy required for anabolism within the alloreactive microenvironment characteristic of GVHD (Mhandire et al., 2021). Furthermore, nicotinamide phosphoribosyltransferase (Nampt), as the rate-limiting enzyme in the NAD salvage pathway, plays a critical role in GVHD. Nampt is highly expressed in the serum of patients with gastrointestinal GVHD and is particularly enriched in T cells of the intestinal tract in both humans and mice (Gerner et al., 2020). Inhibition of Nampt by the small molecule inhibitor FK866 significantly alleviates experimental GVHD and is associated with NAD depletion in T cell subsets, thereby selectively suppressing the proliferation of newly activated T cells while preserving the function of memory T cells (Gerner et al., 2020).

The role of sphingolipid metabolism in GVHD primarily manifests through the regulation of *CerS6* on T-cell migration and activation. *CerS6*, by regulating the synthesis of ceramide platforms, serves as a key regulator in GVHD (Brown and Byersdorfer, 2020). In GVHD, alloreactive T cells utilize various energetic and biosynthetic pathways, which are distinct from the metabolic pathways relied upon by Tregs for their suppressive function. For example, in acute GVHD (aGVHD), T cells primarily rely on glycolysis and oxidative phosphorylation (OXPHOS) for energy. In contrast, during chronic GVHD (cGVHD), T cells may differentiate into IL-21-producing T follicular helper cells (Tfh) or tissue-resident helper T cells, which collaborate with germinal center B cells or memory B cells to produce autoantibodies, leading to tissue fibrosis (Mohamed et al., 2021).

Metabolic intervention strategies have shown promising potential in the treatment of GVHD. For example, inhibition of dihydroorotate dehydrogenase (DHODH) can significantly reduce the severity of GVHD by decreasing the oxidative metabolism of T cells and the production of inflammatory cytokines, while preserving the graft-versus-leukemia (GVL) effect (Kumar et al., 2025). Similarly, targeting 6-phosphogluconate dehydrogenase (6PGD), a key enzyme in the pentose phosphate pathway (PPP), can ameliorate the severity of GVHD by altering the glycolytic dependency of T cells (Daneshmandi et al., 2025). These studies provide a crucial foundation for the development of therapeutic strategies for GVHD based on metabolic regulation.

### 5.2 Research progress on sphingolipid metabolism as a therapeutic target in GVHD

In recent years, the potential role of sphingolipid metabolism in the treatment of GVHD has garnered increasing attention. Studies have demonstrated that the *CerS6* inhibitor ST1072 exhibits significant therapeutic efficacy in both acute and cGVHD models. At the molecular level, *CerS6* regulates T cell function, particularly TCR signaling and PKCθ co-localization in CD4+ T cells, through the modulation of the *N-RAS*/ERK signaling pathway (Sofi et al., 2022). ST1072 not only effectively inhibits the migration of donor T cells to GVHD target organs but also selectively reduces the activation of CD4+ T cells, thereby alleviating GVHD symptoms while preserving anti-tumor activity (GVL effect) (Sofi et al., 2022). This characteristic offers it a distinct advantage over traditional immunosuppressants such as FTY720, which, while capable of mitigating GVHD, concurrently compromises the GVL effect. In cGVHD models, both genetic knockout and pharmacological inhibition of *CerS6* have been shown to effectively prevent and reverse disease progression (Sofi et al., 2022).

Metabolic reprogramming offers a novel approach for the precision treatment of GVHD. Studies have found that human umbilical cord mesenchymal stem cells (HUCMSCs) significantly increase phytosphingosine (PHS) levels by modulating sphingolipid metabolism, thereby ameliorating aGVHD (Hong et al., 2023). PHS inhibits CD4+ T cell proliferation, promotes apoptosis, and reduces Th1 cell differentiation *in vitro*, with its mechanism of action involving the downregulation of pro-inflammatory pathways such as NF-κB (Hong et al., 2023). In animal models, PHS administration significantly ameliorated the development of aGVHD, confirming the potential of sphingolipid metabolites as safe and effective therapeutic strategies (Hong et al., 2023). Furthermore, multi-omics analysis has revealed alterations in the microbiome and metabolome across different intestinal segments and found that metabolites such as sphingolipids are closely associated with gastrointestinal GVHD (Lauder et al., 2025). These findings provide a theoretical basis for the development of targeted therapies for GVHD based on metabolic modulation.

Sphingolipid metabolites may also serve as biomarkers for GVHD. In patients with ocular cGVHD, metabolomic analysis of tear fluid revealed significant dysregulation of the sphingolipid metabolic pathway, with SM and lactosylceramide platforms (LacCer) showing high correlations with clinical parameters such as tear film breakup time and corneal fluorescence staining (Ma et al., 2021). These metabolites not only contribute to disease diagnosis but also, through their dynamic changes, may inform the adjustment of therapeutic regimens. With a deeper understanding of the role of sphingolipid metabolism in T cell responses (Tian et al., 2022), and the ongoing exploration of metabolic reprogramming technologies in the prevention and treatment of GVHD (Kumari et al., 2020), strategies targeting sphingolipid metabolism are poised to become a critical breakthrough in balancing GVHD control with the maintenance of the GVL effect.

## 6 Classic lysosomal storage disorders: prototypical diseases of sphingolipid metabolism dysregulation

### 6.1 Gaucher disease: glucocerebrosidase deficiency and pathological mechanisms

**Figure 8:**
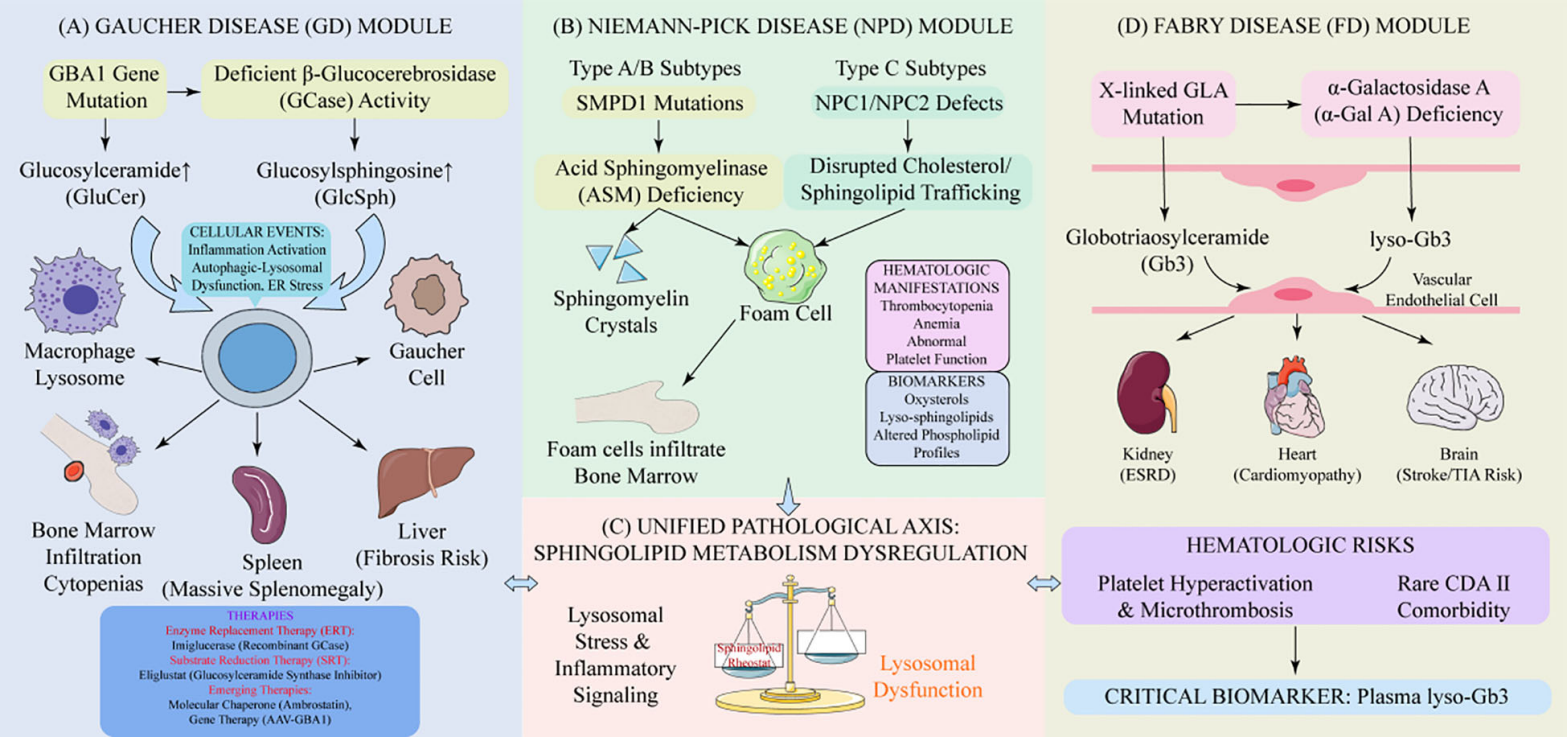
Unified pathological axis of sphingolipid dysregulation in lysosomal storage diseases: Gaucher disease, Niemann-Pick disease, and Fabry disease. This schematic illustrates the shared and distinct mechanisms of sphingolipid metabolic dysregulation in three classical lysosomal storage disorders. The GD module **(A)** highlights *GBA1* gene mutation leading to deficient GCase activity, resulting in accumulation of glucosylceramide platforms (GluCer) and glucosylsphingosine (GlcSph), formation of Gaucher cells, and cellular events including inflammation activation, autophagic-lysosomal dysfunction, and ER stress (Zhao et al., 2023; Méndez-Cobián et al., 2024). Therapeutic interventions for GD include enzyme replacement therapy (e. g., imiglucerase) (Méndez-Cobián et al., 2024), substrate reduction therapy (e. g., eliglustat) (Donald et al., 2022), molecular chaperones (e. g., ambrostatin) (Hughes et al., 2022), and gene therapy (AAV-*GBA1*) (Okai et al., 2025). The NPD module **(B)** depicts type A/B subtypes with SMPD1 mutations causing ASM deficiency and disordered cholesterol/sphingolipid trafficking, leading to SM crystal formation, foam cell infiltration in bone marrow, and hematologic manifestations including neutropenia, thrombocytopenia, and anemia. Relevant biomarkers include cholesterol, lyso-sphingolipids, and altered sphingolipid profiles (Ufuk, 2024; Wu et al., 2024). The Fabry disease (FD) module **(D)** shows X-linked *GLA* mutation causing α-galactosidase A (α-Gal A) deficiency and accumulation of globotriaosylceramide platforms (Gb3) and lyso-Gb3 in vascular endothelial cells (Roy et al., 2024; Podolec et al., 2025). The unified pathological axis **(C)** emphasizes sphingolipid metabolism dysregulation as a convergent mechanism driving lysosomal dysfunction and inflammatory signaling across all three diseases. This schematic incorporates icons from multiple sources: “Healthy_liver” by Jan-Clusmann (CC0 https://creativecommons.org/publicdomain/zero/1.0/); “Spleen-1”, “brain-1”, “kidney-1”, “heart-front”, and “foam-cell-5” by Servier Medical Art (CC-BY 3.0 Unported https://creativecommons.org/licenses/by/3.0/); “Bone_marrow” and “plasma_cell” by El-Jayawant (CC-BY 4.0 Unported https://creativecommons.org/licenses/by/4.0/); and “Mast_cell_1” by Sebastian-Reinig (CC-BY 4.0 Unported https://creativecommons.org/licenses/by/4.0/). All icons were reproduced exactly without modification.

Gaucher disease (GD) is an autosomal recessive lysosomal storage disorder caused by biallelic mutations in the *GBA1* gene, with the core pathological mechanism being a deficiency in the activity of the lysosomal enzyme β-glucocerebrosidase (GCase) (Zhao et al., 2023; Méndez-Cobián et al., 2024) (**Figure 8A**). The GCase enzyme encoded by the *GBA1* gene is responsible for hydrolyzing glucosylceramide (GluCer) into glucose and ceramide platforms (Kim et al., 2024). When GCase function is deficient, its substrate GluCer and its deacylated derivative, glucosylsphingosine (GlcSph), accumulate abnormally in the lysosomes of macrophages, forming characteristic “Gaucher cells” (Horowitz et al., 2022). This molecular process not only leads to lysosomal dysfunction but also triggers a series of secondary pathological events. Gaucher cells primarily infiltrate the bone marrow, spleen, and liver, and this infiltration is the direct cause of bone marrow failure, splenomegaly, bone disease, and abnormal immune activation (Hughes et al., 2019). The infiltration of Gaucher cells in the bone marrow disrupts the normal hematopoietic microenvironment, leading to impaired hematopoiesis and clinically manifesting as anemia, thrombocytopenia, and leukopenia (Özdemir and Gündüz, 2022).

It is critical to note that the primary storage material is GluCer, not ceramide; in fact, the defective hydrolysis of GluCer directly reduces the production of ceramide via the canonical GCase pathway (Arévalo et al., 2022). However, recent studies using stable isotope labeling in a conduritol B epoxide (CBE)-induced GD macrophage model have revealed a compensatory upregulation of the *de novo* sphingolipid synthesis pathway (Lake et al., 2025). Specifically, this study found increased labeling of C16:0 ceramide, while C24:0 ceramide remained unchanged or decreased. The authors propose that this upregulation represents a compensatory mechanism by GD macrophages to maintain ceramide homeostasis, offsetting the loss of ceramide derived from the impaired GlcCer degradation pathway. This suggests that while the degradative pathway produces less ceramide, cells may enhance *de novo* synthesis to “compensate,” resulting in a paradoxical increase in certain ceramide species (e.g., short-chain C16:0) (Lake et al., 2025).

Therefore, the net change in ceramide levels in GD is heterogeneous and context-dependent. At the whole-organism or tissue level, a net decrease may be observed due to the primary degradative block. However, within macrophages, a secondary, compensatory elevation of specific ceramide species may occur. This secondary elevation is not directly driven by substrate accumulation but represents a metabolic adaptation by the cell to a perceived ceramide deficiency. It is critical to distinguish this secondary ceramide elevation from the primary accumulation of GluCer and GlcSph. Furthermore, this dysregulation extends beyond ceramide to impact the broader sphingolipid signaling network, including the S1P axis (Indellicato and Trinchera, 2019).

Disorders of sphingolipid metabolism are considered a key factor driving GD complications. Studies have shown that the accumulation of GluCer and GlcSph triggers chronic inflammatory responses and immune activation (Miano et al., 2020).Furthermore, the imbalance in sphingolipid metabolism also affects cellular functions, such as leading to dysfunction of the autophagy-lysosome pathway (Kinghorn et al., 2017)and neuroinflammation (e.g., microgliosis) (Zhao et al., 2023),and may involve processes like the unfolded protein response (endoplasmic reticulum stress) (Horowitz et al., 2022).Together, these events exacerbate cellular damage and tissue dysfunction (Meng et al., 2025).Therefore, the pathological manifestations of Gaucher disease extend far beyond simple substrate accumulation, involving complex dysregulation of cellular metabolic and signaling pathways.

The treatment strategies for Gaucher disease focus on correcting lipid metabolism disorders, with primary approaches including Enzyme Replacement Therapy (ERT) and Substrate Reduction Therapy (SRT). Enzyme Replacement Therapy (ERT) involves the intravenous infusion of recombinant human GCase (such as imiglucerase or velaglucerase alfa), which is taken up by macrophages and supplements the deficient enzyme activity in their lysosomes, thereby degrading accumulated GluCer (Méndez-Cobián et al., 2024). ERT effectively ameliorates hepatosplenomegaly, corrects cytopenias, and alleviates some bone pain; however, it is unable to cross the blood-brain barrier and is therefore ineffective for the central nervous system symptoms of neuronopathic Gaucher disease (nGD) (Donald et al., 2022). Substrate Reduction Therapy (SRT) employs orally administered small molecule drugs (such as Eliglustat), which work by inhibiting glucosylceramide platforms synthase to reduce the *de novo* synthesis of GluCer, thereby decreasing its lysosomal burden (Ain et al., 2025). SRT can also improve systemic symptoms and offers the advantage of oral administration, but its neuroprotective effects in nGD are limited (Harai et al., 2023). Emerging chaperone therapy aims to use small molecules (such as ambroxol) to stabilize misfolded mutant GCase, promoting its correct folding and trafficking to the lysosome, thereby restoring partial enzyme activity; this approach is currently still under investigation (Hughes et al., 2022). Gene therapy represents another promising direction, aimed at achieving long-term or even permanent expression of GCase by delivering a functional *GBA1* gene via viral vectors such as AAV. Preclinical studies have demonstrated that AAV-mediated delivery of *GBA1* not only reduces GlcSph levels and improves motor deficits in GD model mice but also decreases α-synuclein aggregation, offering an additional benefit for GD patients with comorbid Parkinson’s disease risk (Okai et al., 2025).

However, during clinical promotion and long-term application, a series of issues and challenges have gradually emerged, such as high treatment costs limiting accessibility, treatment resistance or efficacy decline in some patients, difficulty in reversing neurological symptoms, and safety and ethical controversies surrounding gene therapy (Camou and Berger, 2025). These issues not only affect patient treatment outcomes but also pose significant challenges to the allocation of medical resources and public health policies. Therefore, a thorough analysis of the challenges and issues in the treatment of Gaucher disease is crucial for deepening our understanding of sphingolipid metabolism and advancing precision medicine (Grabowski et al., 2025). Future research should focus on enhancing the efficiency and safety of sphingolipid metabolic regulation to improve patients’ quality of life and disease management.

### 6.2 Niemann-Pick disease and Fabry disease: distinct enzyme deficiencies and hematological manifestations

Niemann-Pick disease (NPD) (**Figure 8B**) and Fabry disease (FD) (**Figure 8D**) are two other important lysosomal storage disorders involving sphingolipid metabolism dysregulation. They are caused by distinct enzyme deficiencies and exhibit unique hematological and multisystem involvement characteristics. Niemann-Pick disease is primarily classified into types A/B and type C. Types A and B are caused by a deficiency in acid sphingomyelinase (ASM), leading to the accumulation of SM within the mononuclear-phagocyte system (Wu et al., 2024). Type C is predominantly caused by mutations in the *NPC1* or *NPC2* genes, which impair the transport of cholesterol and sphingolipids (such as SM and glucosylceramide platforms) out of late endosomes/lysosome (Ufuk, 2024). In NPD, lipid-laden macrophages (i. e., “foam cells”) infiltrate the bone marrow, disrupting the hematopoietic microenvironment. This can lead to impaired hematopoiesis, manifesting as peripheral blood cytopenias, such as thrombocytopenia and anemia (Qadir et al., 2021). In a mouse model of *NPC1* deficiency, an increase in platelet count, impaired aggregation function, and prolonged bleeding time were also observed, suggesting a defect in the function of platelet lysosome-related organelles (Chen et al., 2020). Collectively, these alterations affect the hematopoietic function of the bone marrow and the normal physiology of blood cells.

Fabry disease is an X-linked genetic disorder caused by mutations in the *GLA* gene, leading to a deficiency in the activity of α-galactosidase A (α-Gal A). This deficiency results in the accumulation of globotriaosylceramide platforms (Gb3) and its deacylated form, lyso-Gb3, in the lysosomes of various cell types (Podolec et al., 2025). Although Fabry disease primarily affects the kidneys, heart, and nervous system, its association with the hematological system is increasingly being recognized. Vascular endothelial cells are a primary target for Gb3 accumulation, and their damage can lead to microangiopathy and an increased risk of thrombosis (Roy et al., 2024). Additionally, a case report has described the rare coexistence of Fabry disease with congenital dyserythropoietic anemia (CDA) type II, suggesting a potential for broader hematopoietic system involvement (Elsherif et al., 2024).

The specific alterations in the sphingolipid profile observed in these diseases offer potential as biomarkers for diagnosis and therapeutic monitoring. In Fabry disease, the plasma level of lyso-Gb3 has been established as an important biomarker for the classic phenotype, serving to aid in diagnosis, assess disease severity, and monitor therapeutic response (Simonetta et al., 2020). In Niemann-Pick disease type C, in addition to classic biomarkers such as oxysterols and lyso-sphingolipids, comprehensive lipidomic analysis has revealed specific alterations in phospholipids and sphingolipids, providing a distinct biosignature for the disease (Boenzi et al., 2021). For Gaucher disease, in addition to the traditional biomarker chitotriosidase, emerging biomarkers such as plasma GlcSph and glycoprotein non-metastatic melanoma protein B (gpNMB) have demonstrated improved specificity and correlation with disease activity (Marano et al., 2024). The quantitative analysis of these specific sphingolipids and their metabolites using techniques such as mass spectrometry not only facilitates early and precise diagnosis but also enables the dynamic monitoring of substrate clearance during treatment, allowing for the evaluation of the efficacy of enzyme replacement therapy or substrate reduction therapy. This, in turn, provides a basis for the adjustment of individualized treatment regimens (Mashima et al., 2020). But clinical applicability of these biomarkers varies. Lyso-Gb3 is widely used for Fabry disease, yet false negatives occur in female heterozygotes (Ramaswami et al., 2025). For Niemann-Pick type C, the reported lipidomic signatures have not been validated in independent cohorts (Boenzi et al., 2021). In Gaucher disease, GlcSph and gpNMB show promise, but standardized assays and cut-off values are still lacking (Brody et al., 2024). Most of these biomarkers remain without multi-center validation or routine quality control. So their diagnostic and monitoring value, while promising, is not fully established. Therefore, a thorough understanding of the specific dysregulations of sphingolipid metabolism in different lysosomal storage disorders is essential for the development and application of effective biomarker systems.

## 7 The emerging role of sphingolipid metabolism in thalassemia and sickle cell disease

**Figure 9:**
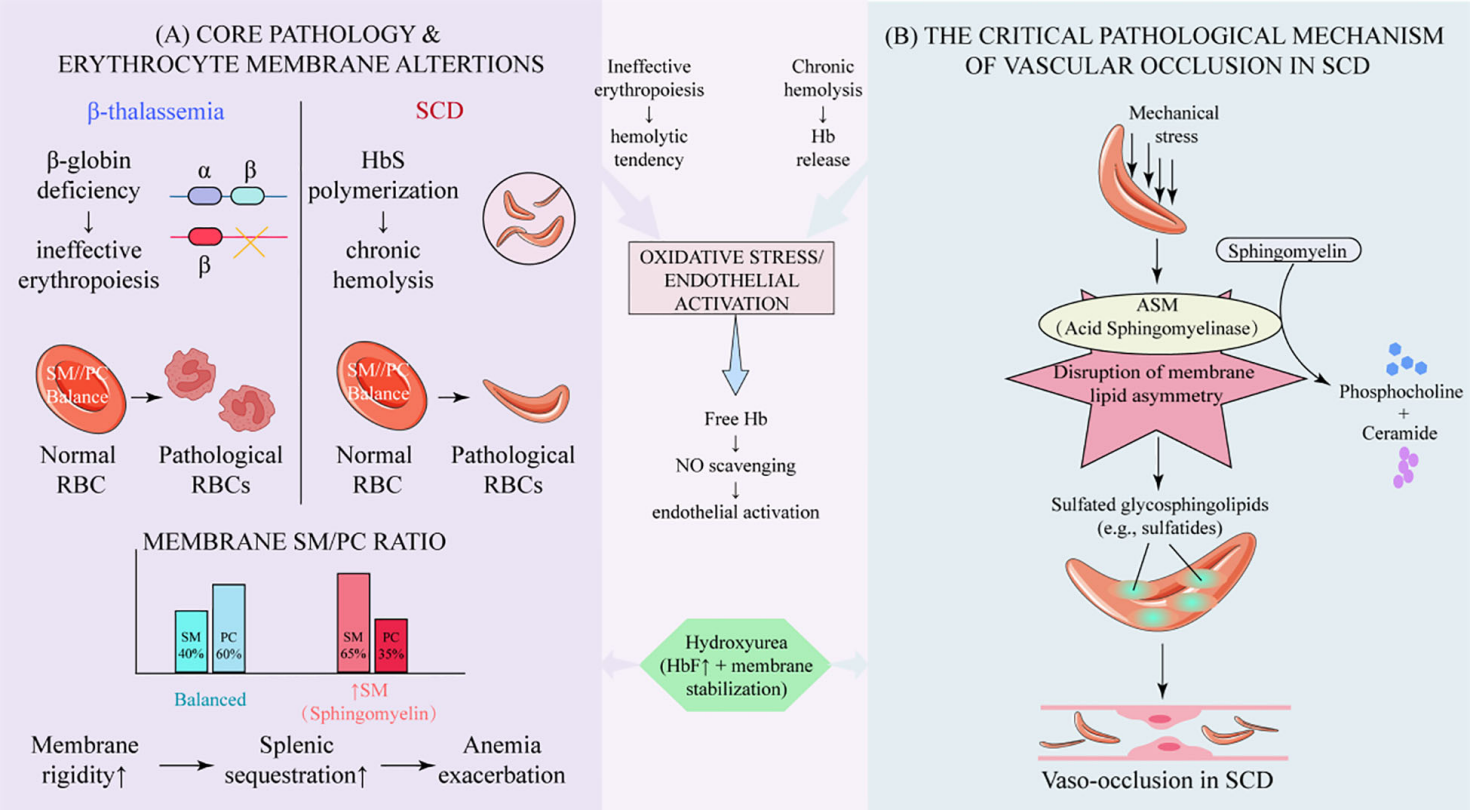
Shared pathogenic role of sphingolipid metabolic dysregulation in β-thalassemia and sickle cell disease. Description: This schematic illustrates the convergent mechanisms of sphingolipid metabolism dysregulation in β-thalassemia and SCD, highlighting their shared pathway toward hemolytic complications and organ damage. The panel **(A)** (Core Pathology & Erythrocyte Membrane Alterations) demonstrates disease-specific initiating events: β-globin deficiency leading to ineffective erythropoiesis in β-thalassemia, and hemoglobin S polymerization causing chronic hemolysis in SCD (Lopes et al., 2022). Both conditions result in altered erythrocyte membrane composition with increased SM/PC ratio, reducing membrane fluidity and deformability (Lopes et al., 2022). The central panel (Oxidative Stress/Endothelial Activation) shows how chronic hemolysis and hemoglobin release lead to nitric oxide (NO) scavenging, oxidative stress, and endothelial activation (Risoluti et al., 2020). The panel **(B)** illustrates the critical pathological mechanism of vascular occlusion in SCD (Goreke et al., 2023). This schematic incorporates the following icons from Servier Medical Art: “Falciform-erythrocyte-2” and “erythrocyte-1” (CC-BY 3.0 Unported https://creativecommons.org/licenses/by/3.0/), adapted with modifications.

β-thalassemia and SCD, as the most common monogenic diseases worldwide, have pathological processes that are closely associated with sphingolipid metabolic reprogramming (Leonard et al., 2022) (**Figure 9**). In β-thalassemia, insufficient synthesis of β-globin leads to ineffective erythropoiesis, while in SCD, the polymerization of hemoglobin S triggers chronic hemolysis. Both of these pathological conditions significantly alter the sphingolipid composition of the erythrocyte membrane. Studies have shown that the ratio of SM to PC is abnormally elevated in the erythrocyte membranes of patients, and this alteration directly impacts membrane fluidity and mechanical stability (Lopes et al., 2022). Consequently, when the SM/PC ratio falls outside the physiological range, erythrocyte deformability is compromised, making the cells more susceptible to being sequestered and cleared by the spleen, thereby shortening the erythrocyte lifespan and exacerbating anemic symptoms.

Research indicates that in erythrocytes of β-thalassemia heterozygotes, sphingomyelin levels are significantly reduced, accompanied by disturbances in redox metabolism (Nuinoon et al., 2026). The externalization of phosphatidylserine on the erythrocyte surface—a hallmark of eryptosis—is markedly increased in thalassemia patients, further confirming the critical role of membrane lipid dysregulation in the pathological process (Kargutkar and Nadkarni, 2023). Additionally, the fetal hemoglobin (HbF) inducer hydroxyurea, while elevating HbF levels, has been found to modulate the erythrocyte membrane sphingolipid composition, which may represent an additional mechanism contributing to its clinical benefits (Therrell, 2025).

In SCD, chronic hemolysis and hypoxia-driven polymerization of HbS impose mechanical bending stress on the erythrocyte membrane. The accumulated energy activates acid sphingomyelinase, leading to sphingomyelin hydrolysis and subsequent exposure on the outer membrane leaflet (Goreke et al., 2023). These surface-exposed sulfatides mediate abnormal, hypoxia-enhanced adhesion of mature sickle erythrocytes to laminin and the vascular endothelium—a key step in vaso-occlusive crises (Goreke et al., 2023).

However, there is a severe lack of large-scale, longitudinal clinical sphingolipidomics data in patients with hemoglobinopathies. Although studies have revealed associations between specific ceramide and sphingomyelin species in the plasma of SCD patients and multi-site vaso-occlusive crises (Alp et al., 2024), these are predominantly cross-sectional investigations, making it difficult to establish a causal relationship between specific sphingolipid species and long-term clinical outcomes.

## 8 Future directions and clinical application prospects in sphingolipid metabolism research

### 8.1 Multi-omics integration and precision medicine

By integrating metabolomics, genomics, and proteomics data, researchers can now systematically reveal the dynamic alterations of the sphingolipid metabolic network during disease onset and progression. For example, a multi-omics study focusing on erythroid differentiation revealed that the hematopoietic transcription factor GATA1 establishes ceramide platforms homeostasis by regulating the expression of sphingolipid metabolic enzymes, such as Δ4-desaturase. This process is critical for erythroid progenitor cell viability but has no significant effect on myeloid progenitors (Liao et al., 2023). This provides a theoretical basis and technical support for the personalized treatment of lipid metabolism-related hematological disorders.

Future directions must address the technical challenges of multi-omics data integration, including the standardization of heterogeneous data, the interpretability of algorithms, and the optimization of clinical translation pathways. For instance, a recently developed lipid-metabolite-protein network framework utilizes hyperbolic embedding techniques to visualize the functional proximity of molecules across different omics layers, providing a platform for the discovery of novel biomarkers associated with sphingolipid metabolism, such as 4-imidazoleacetic acid (Anyaegbunam et al., 2025). Concurrently, the integration of functional precision medicine (e. g., drug screening using patient-derived organoids) with multi-omics approaches holds promise for overcoming the limitations of traditional molecular subtyping and for providing more precise therapeutic strategies for hematological diseases associated with sphingolipid metabolism abnormalities (Zhang et al., 2025).

### 8.2 Development and validation of novel therapeutic targets

As a key pathway in cellular signal transduction and membrane structure regulation, sphingolipid metabolism has shown significant potential in the development of therapeutic targets for hematological diseases in recent years. The development of inhibitors targeting key enzymes in sphingolipid metabolism has become an important direction in this field, with particularly notable progress in research on *CerS6* inhibitors. In mouse models, specific inhibition of *CerS6* not only effectively prevents and reverses cGVHD but also alleviates aGVHD symptoms, while preserving the graft-versus-leukemia (GVL) effect—a characteristic that offers a significant advantage over the immunosuppressant FTY720 (Sofi et al., 2022). At the molecular level, *CerS6* selectively activates CD4+ T cells by promoting the co-localization of CD3/PKCθ and enhancing TCR signaling, a process that can be blocked by small molecule inhibitors of *CerS6* such as ST1072 (Sofi et al., 2022). Furthermore, based on structure-based virtual screening techniques, researchers have identified a novel *CERS2* inhibitor, Hit-325144, from the ZINC20 database. This compound exhibits a dose-dependent inhibitory capacity on enzyme activity by stably binding to the His212/His213 residues in the catalytic site of *CERS2*, offering a new avenue for targeted therapy in metabolic and cardiovascular diseases (Yu et al., 2026). These findings provide a theoretical foundation for the development of precision therapeutic strategies targeting sphingolipid-metabolizing enzymes.

Targeted intervention of the ceramide metabolic pathway represents a cutting-edge direction in the current treatment of hematologic malignancies. Studies have demonstrated that in drug-resistant AML cells, simultaneous inhibition of ceramide hydrolysis (using SACLAC) and glucosylation (using D-threo-PDMP) results in synergistic cytotoxicity, significantly increasing all molecular species of ceramide, and inducing mitochondrial respiratory dysfunction and caspase-driven cell death (Fisher-Wellman et al., 2023). Furthermore, resveratrol exerts antiproliferative and pro-apoptotic effects in FLT3-ITD-positive AML cells by downregulating the expression of sphingosine kinase-1 (SK-1) and glucosylceramide synthase (GCS) (Ersöz and Adan, 2022). In CLL, the GCS inhibitor eliglustat, which is already approved for Gaucher disease, significantly reduces cell viability and displays synergy with ibrutinib (Nguyen Van Long et al., 2024). Collectively, these findings indicate that blocking ceramide clearance pathways through multiple strategies is a promising approach to overcome drug resistance in hematologic malignancies.

### 8.3 Dynamic alterations of sphingolipid metabolism in transfused erythrocytes and their clinical implications

The dynamic changes in sphingolipid metabolism of erythrocytes during storage exert a significant impact on the efficacy of blood transfusions. Studies have shown that with prolonged storage time, the sphingolipid composition of the erythrocyte membrane undergoes significant alterations, characterized by the abnormal accumulation of lipid molecules such as SM and dihydrosphingomyelin (DHSM) (Kinoshita et al., 2020). This metabolic dysregulation is closely associated with the remodeling of erythrocyte membrane microdomains (lipid rafts), in which DHSM forms unique molecular aggregates within the SM-enriched ordered domains, significantly enhancing the order of membrane lipids (Kinoshita et al., 2020). This alteration leads to decreased membrane fluidity of erythrocytes, thereby influencing the distribution and function of the Piezo1 mechanosensitive ion channel (Stommen et al., 2023). This suggests that assessing the sphingolipid metabolic status of erythrocytes prior to transfusion is of significant importance for improving patient outcomes.

The disruption of sphingolipid metabolism caused by storage lesion affects the safety of blood transfusions through multiple mechanisms. First, prolonged storage leads to the accumulation of sphingolipid metabolites, such as ceramide platforms, in erythrocytes. These molecules, functioning as second messengers, can activate inflammatory signaling pathways (Bal et al., 2026). Clinical data indicate that the supernatant from erythrocytes stored for 42 days can significantly promote mast cell degranulation, thereby increasing the risk of allergic transfusion reactions (Bal et al., 2026). On the other hand, in patients with SCD, transfusion of erythrocytes stored for prolonged periods (≥30 days) results in significantly elevated plasma levels of oxidative stress markers and pro-inflammatory cytokines, accompanied by an increase in markers of renal injury (Karafin et al., 2025). Metabolomics analysis has revealed that these alterations are closely related to abnormalities in the sphingolipid metabolic pathway, and notably, the metabolic reprogramming mediated by *CerS6* plays a critical role in the development of GVHD (Sofi et al., 2022).

Optimizing transfusion strategies requires a comprehensive assessment of the RBC sphingolipid metabolic network, particularly the dynamic balance of its core metabolite, S1P, and its intricate interplay with membrane structure and redox status. Studies have demonstrated that plasma S1P levels in anemic patients show a strong linear positive correlation with hematocrit, yet standard RBC transfusions do not consistently restore plasma S1P levels. This limitation is especially pronounced when transfusing RBCs stored for extended periods, as their internal S1P content declines to as low as 19% of fresh units, severely limiting their capacity to replenish circulating S1P (Selim et al., 2011). Beyond depleting endogenous RBC S1P, storage profoundly alters RBC membrane lipid composition, lateral organization, and biophysical properties. For instance, storage induces dynamic changes in cholesterol- and sphingomyelinase-associated lipid domains, which interact with intracellular calcium influx and oxidative stress accumulation to drive extracellular vesicle biogenesis (Cloos et al., 2020). This complex membrane remodeling further disrupts S1P metabolism and signaling. Although exogenous S1P supplementation can promote glycolysis and ATP generation in stored RBCs, it does so at the cost of inhibiting the pentose phosphate pathway and exacerbating oxidative damage, ultimately reducing post-transfusion recovery (Hay et al., 2023). Moreover, donor characteristics (e.g., age, sex) affect baseline RBC S1P levels (Hay et al., 2023), while preparation methods (e.g., platelet concentrates exhibit significant S1P decline during storage (Poppe et al., 2019)) and recipient pathological conditions (e.g., ASM activation in sickle cell disease, leading to increased S1P levels and pro-inflammatory microparticles (Awojoodu et al., 2014); or critically ill patients with already low plasma S1P that transiently declines further after transfusion (Poppe et al., 2019)) significantly modulate sphingolipid-mediated physiological responses. Therefore, future transfusion optimization should move beyond simple S1P supplementation. Instead, a comprehensive approach that integrates blood product source, storage duration, storage conditions (e.g., hypoxic storage (Hay et al., 2023)), and individual recipient pathophysiological features is needed. By precisely regulating the entire sphingolipid metabolic axis, it may be possible to simultaneously enhance RBC energy status, maintain membrane integrity, and preserve redox homeostasis, thereby improving transfusion efficacy and clinical outcomes (Hay et al., 2023).

### 8.4 Innovations in clinical diagnostic and monitoring tools

Sphingolipid metabolites, as biomarkers, show significant potential in the diagnosis and therapeutic monitoring of hematological diseases. In recent years, significant progress has been made in mass spectrometry-based sphingolipidomics analysis methods, providing new insights for the development of non-invasive diagnostic tools. Studies have shown that liquid chromatography-tandem mass spectrometry (LC-MS/MS) technology can simultaneously detect 47 sphingolipid species related to ceramide platforms metabolism, with a linear range (r² > 0. 99) and recovery rates (80%-120%) that meet clinical testing standards (Byeon et al., 2024).

However, technical innovation and clinical translation continue to face challenges. Current methods for sphingolipid detection need to address three key issues: First, standardization—the detection results for the same sphingolipid (e. g., C24:0 ceramide platforms) can differ by up to 30% across different research centers (Mah et al., 2021);Second, dynamic range limitations—the circulating concentration of low-abundance sphingolipids (e. g., sphingosine-1-phosphate) is often below 1 μM, necessitating the combination with ultra-performance liquid chromatography (UPLC) to enhance sensitivity (Esfandiary et al., 2022); Third, bioinformatics analysis—although a panel of 15 characteristic sphingolipids identified via partial least squares discriminant analysis (PLS-DA) can distinguish patients with metabolic syndrome (VIP > 1. 0), the multiple linear regression model integrating these features with clinical parameters such as waist circumference and insulin resistance still requires validation in large-scale cohorts (Rigamonti et al., 2023). Future directions include the development of microfluidic chips for point-of-care testing, the establishment of sphingolipid metabolism databases to support AI-assisted diagnosis, and the exploration of exosomal sphingolipid biomarkers for monitoring minimal residual disease (MRD) (Obeagu, 2025). These innovative tools are poised to drive a paradigm shift in the management of hematological diseases, transitioning from traditional morphological diagnosis toward molecular subtyping and real-time, dynamic management.

### 8.5 Personalized treatment through metabolic intervention

Abnormalities in sphingolipid metabolism play a critical role in the initiation, progression, and treatment response of hematologic malignancies, providing a robust theoretical foundation for personalized therapeutic strategies tailored to the specific metabolic characteristics of individual patients. In AML, studies have revealed that inhibiting sphingosine kinase induces the accumulation of ceramide platforms, which in turn activates the integrated stress response mediated by protein kinase R. This activation subsequently upregulates the pro-apoptotic protein Noxa and degrades the pro-survival protein Mcl-1, on which AML cells are highly dependent (Lewis et al., 2022). This mechanism not only elucidates the anticancer role of ceramide platforms but, more importantly, intervention targeting this pathway synergizes with the Bcl-2 inhibitor venetoclax to effectively kill primary AML blasts—including those resistant to venetoclax—as well as immunophenotypic leukemia stem cells. Furthermore, this combination strategy has been shown to impair leukemia engraftment in patient-derived xenograft models (Lewis et al., 2022). This suggests that designing regimens that co-target Bcl-2 and sphingolipid metabolic pathways, based on the dependence of AML tumor cells on Mcl-1 and their sphingolipid metabolic status, holds promise for overcoming drug resistance and achieving precise treatment. Similarly, in chronic lymphocytic leukemia (CLL), untargeted metabolomics and lipidomics analyses have revealed distinct signatures of sphingolipid dysregulation associated with aggressive CLL and poor survival outcomes (Nguyen Van Long et al., 2023). Specifically, the expression levels of multiple genes encoding enzymes in the sphingolipid biosynthesis pathway were found to be significantly associated with shorter patient survival. Furthermore, specific circulating sphingolipid species, such as glucosylceramide platforms (C16:0 GluCer) and sphingosine, were identified as independent prognostic markers, exerting opposing pro-proliferative and pro-apoptotic effects, respectively (Nguyen Van Long et al., 2023). Therefore, risk stratification regarding disease invasiveness and prognosis can be achieved by detecting the levels of these sphingolipids in patient plasma. Building on this, the use of an inhibitor of glucosylceramide platforms synthase to block GluCer synthesis was successfully used to induce cell death and reduce the proliferative phenotype in leukemic B-CLL cell models, while simultaneously enhancing their sensitivity to anti-leukemic drugs such as fludarabine and ibrutinib (Nguyen Van Long et al., 2023). This provides a direct rationale for developing combination treatment regimens that incorporate metabolic intervention for CLL patients with specific sphingolipid profiles, such as those exhibiting high GluCer levels. More broadly, lipid metabolic reprogramming in B-cell malignancies, including the dysregulation of sphingolipid metabolism, is recognized as one of the key intrinsic and extrinsic factors driving their malignant phenotype (Lee et al., 2024). Targeting these altered lipid metabolic pathways holds the translational potential to improve risk stratification and clinical management across different subtypes of B-cell malignancies (Lee et al., 2024). The core of achieving personalized metabolic intervention lies at the heart of accurately assessing a patient’s metabolic profile. This necessitates the integration of multi-omics data, such as metabolomics, lipidomics, and transcriptomics, to map the patient-specific metabolic landscape. Simultaneously, consideration must be given to the social determinants of health and chronic physiological stress loads that influence metabolic status. For example, in chronic myeloid leukemia (CML), a higher allostatic load is associated with adverse social determinants of health and independently predicts poorer treatment response and outcomes (Miranda-Galvis et al., 2025). Therefore, future personalized treatment will require not only targeting the intrinsic metabolic vulnerabilities of tumor cells but also incorporating the patient’s holistic metabolic and physiological stress status into consideration. Through multidisciplinary collaboration, comprehensive and personalized metabolic intervention strategies should be developed, with the aim of enhancing therapeutic efficacy while minimizing treatment-related toxicity and improving patient quality of life.

### 8.6 Adipose metabolism biomarkers: a summary from mechanism to clinical application

Based on the aforementioned discussion, abnormalities in the sphingolipid metabolic network have identified a series of biomarkers with potential diagnostic, prognostic, or therapeutic monitoring value in various hematologic disorders. The table below provides a systematic summary and evaluation of these biomarkers, aiming to offer a clear reference for future clinical translational research. **Table 1**.

**Table 1:** Summary and evaluation of biomarkers associated with sphingolipid metabolism.

Disease	Biomarker	Source/sample type	Diagnostic or prognostic value	References and notes
Immune Thrombocytopenia (ITP)	Sphingolipid metabolism pathway (overall)	Bone marrow	Prognostic/Predictive of treatment response	Machine learning models (e.g., Boruta algorithm, random forest) can differentiate responses to various treatments (e.g., corticosteroids, TPO-RAs). This is a composite marker, not a single molecule (Li et al., 2024).
	Taurine	Spleen tissue	Diagnostic & Prognostic	Diagnostic: Highly sensitive and specific marker to distinguish ITP spleen from normal spleen. Prognostic: Levels are negatively correlated with splenectomy efficacy (r = -0.52, p < 0.003), serving as a predictive indicator of surgical response (Wen et al., 2022; Huang et al., 2026).
	Other spleen metabolites (Alanine, Glutamine, Myo-inositol, GPC)	Spleen tissue	Diagnostic	Significantly altered compared to normal spleen, forming a splenic metabolic signature for ITP (Wen et al., 2022).
	Various lipids (long-chain fatty acids, oxidized lipids, glycerophospholipids, etc.)	Bone marrow	Predictive of treatment response	Used to distinguish responses to different drugs, enabling personalized treatment (Li et al., 2024).
Fabry Disease	Lyso-Gb3 (globotriaosylsphingosine)	Plasma	Diagnostic & Therapeutic monitoring	Diagnostic: Well-established important biomarker for the classic phenotype, aiding in diagnosis and disease severity assessment. False negatives can occur in female heterozygotes. Monitoring: Used for dynamic monitoring of substrate clearance and therapeutic efficacy (Mashima et al., 2020; Simonetta et al., 2020; Ramaswami et al., 2025).
	Gb3 (globotriaosylceramide)	Vascular endothelial cells, etc.	Auxiliary diagnosis	Its accumulation is the core pathological feature of Fabry disease (Roy et al., 2024; Podolec et al., 2025).
Gaucher Disease	GlcSph (glucosylsphingosine)	Plasma	Diagnostic & Monitoring	Diagnostic: Emerging biomarker with improved specificity over traditional markers (e.g., chitotriosidase) and correlation with disease activity. Monitoring: Used to evaluate the efficacy of enzyme replacement or substrate reduction therapy. Note: standardized assays and cutoff values are still lacking (Brody et al., 2024; Marano et al., 2024).
	gpNMB (glycoprotein non-metastatic melanoma protein B)	Plasma	Monitoring	Emerging biomarker, shows promise for correlation with disease activity. Note: standardized assays and cutoff values are still lacking (Brody et al., 2024; Marano et al., 2024).
Multiple Myeloma	S1P (sphingosine-1-phosphate)	Tumor cells/Tumor microenvironment	Prognostic & Therapeutic target	GFI1 suppresses SGPP1, leading to S1P accumulation and promoting tumor cell survival (p53-independent). High S1P levels may correlate with poor prognosis (Petrusca et al., 2022b).
Chronic Myeloid Leukemia	S1P/SK1 (sphingosine kinase 1)	Tumor cells	Therapeutic resistance	BCR-ABL activates SK1 via the PI3K/AKT pathway, converting pro-apoptotic ceramide into pro-survival S1P, an important mechanism of treatment resistance (Gao et al., 2022).
Sickle Cell Disease	Sulfatide (exposure)	Red blood cell membrane	Pathology/Prognostic	Under hypoxic conditions, exposure of sulfatides on the RBC membrane mediates abnormal RBC-vascular endothelial adhesion, a key step in vaso-occlusive crises (Goreke et al., 2023).
	Plasma sphingolipids (e.g., specific ceramides)	Plasma	Prognostic	Cross-sectional studies suggest associations between certain plasma sphingolipid species (e.g., C16:0 ceramide) and multi-site vaso-occlusive crises (Alp et al., 2024).
β-Thalassemia	SM/PC ratio (sphingomyelin/phosphatidylcholine)	Red blood cell membrane	Pathology	The ratio is abnormally elevated in the RBC membrane, reducing membrane fluidity and deformability, shortening the RBC lifespan (Lopes et al., 2022; Nuinoon et al., 2026).
Graft-Versus-Host Disease (GVHD)	SM (sphingomyelin), LacCer (lactosylceramide)	Tear fluid	Diagnostic	Significantly dysregulated in ocular chronic GVHD, highly correlated with clinical parameters (e.g., tear film breakup time, corneal fluorescein staining). A potential non-invasive diagnostic biomarker (Ma et al., 2021).
	Phytosphingosine (PHS)	Serum/Microenvironment	Therapeutic/Protective	Human umbilical cord mesenchymal stem cells elevate PHS levels to inhibit pro-inflammatory T-cell responses, ameliorating GVHD. A marker of therapeutic potential, not diagnosis (Hong et al., 2023).

## 9 Conclusion

As a crucial component of cellular signal transduction and biofilm structure, sphingolipid metabolism plays a pivotal role in the regulation of hematological diseases, a function that has been extensively validated. From an expert perspective, current research has preliminarily established a conceptual framework linking the sphingolipid metabolic network with the pathogenesis and progression of hematological diseases, elucidating its multifaceted biological effects on immune regulation, cell proliferation, inflammatory responses, and oxygenation function. This understanding not only expands our comprehension of disease pathogenesis but also provides new insights for clinical diagnosis and treatment.

A significant breakthrough in recent years has been the integrated application of metabolomics and machine learning technologies. By combining high-throughput detection technologies with artificial intelligence algorithms, researchers are able to identify sphingolipid biomarkers with diagnostic and prognostic value from within complex metabolic networks. This method not only enhances the accuracy of early disease diagnosis but also provides an objective basis for predicting treatment response. However, it is important to note that the biomarker profiles reported across different studies exhibit certain discrepancies, which may stem from heterogeneity in sample sources, detection methods, and data analysis workflows. Future research needs to establish standardized detection and analysis workflows and enhance the reproducibility and value for clinical translation of research findings through multi-center validation.

Regarding therapeutic targets, the discovery of key enzymes in sphingolipid metabolism, such as *CerS6*, offers a novel direction for intervention in disease treatment. Particularly in refractory conditions such as graft-versus-host disease, therapeutic strategies targeting sphingolipid metabolism have demonstrated unique advantages. It should be noted, however, that existing research is largely confined to cellular and animal models, and clinical translation continues to face numerous challenges, including drug delivery efficiency, targeting specificity, and adverse effects. Furthermore, evaluations of the therapeutic effects targeting the same molecule may differ across studies, a discrepancy that may be attributed to disease heterogeneity, the selection of animal models, and the use of different evaluation criteria. Therefore, future research needs to strengthen the connection between preclinical and clinical studies, develop animal models that more closely mimic human diseases, and implement rigorous clinical trial designs.

Looking ahead, research on sphingolipid metabolism needs to advance toward multi-omics integration and precision medicine. On one hand, by integrating genomics, transcriptomics, proteomics, and metabolomics data, a more comprehensive understanding of the regulatory networks of sphingolipid metabolism in hematological diseases can be achieved. On the other hand, precision diagnostic and therapeutic strategies based on individualized metabolic profiles will contribute to improving treatment outcomes and reducing adverse effects. Additionally, it is essential to strengthen the dialogue between basic research and clinical application, establish translational research platforms, and facilitate the clinical application of research findings. Furthermore, given the complexity and individual variability of sphingolipid metabolism, future therapeutic strategies may require the adoption of combination targeting or multi-target interventions to achieve optimal treatment outcomes.

In conclusion, research on sphingolipid metabolism has opened new avenues for the diagnosis and treatment of hematological diseases; however, numerous scientific questions and technical challenges remain to be addressed. Through interdisciplinary collaboration and the in-depth advancement of translational research, the regulation of sphingolipid metabolism is poised to become an important component of precision medicine for hematological diseases.
